# Neuroimmune circuits in respiratory pathophysiology: decoding molecular crosstalk for precision therapeutic targeting

**DOI:** 10.1080/07853890.2026.2620337

**Published:** 2026-01-27

**Authors:** Qian Chen, Nan Jia, Junling Liu, Caiyou Xu, Zherui Shen, Shihua Shi, Fei Wang, Zhenxing Wang

**Affiliations:** ^a^ Hospital of Chengdu University of Traditional Chinese Medicine, Chengdu, China; ^b^ First School of Clinical Medicine, Nanjing University of Traditional Chinese Medicine, Nanjing, China; ^c^ Friedrich Miescher Institute for Biomedical Research (FMI), Basel, Switzerland

**Keywords:** Neuroimmune axis, bidirectional crosstalk, respiratory diseases, precision medicine, translational therapeutics

## Abstract

**Background:**

The lung is a uniquely innervated barrier organ with dynamic neuroimmune interactions that critically regulate respiratory homeostasis. Despite the well-characterized gut-brain axis, pulmonary neuroimmune crosstalk remains an underexplored frontier. Emerging evidence implicates dysregulated neuron-immune dialogues as key drivers of respiratory pathologies, yet systematic dissection of their bidirectional mechanisms – from neurogenic immunomodulation to immune-mediated neural plasticity – is lacking.

**Main body:**

This review establishes a hierarchical framework for pulmonary neuroimmune crosstalk. We first delineate the lung’s specialized neuroimmune architecture, then synthesize cutting-edge evidence on neuron-immune dialogues across five major respiratory diseases: pulmonary infections, asthma, chronic obstructive pulmonary disease, pulmonary fibrosis and lung cancer. Notably, we identify feedback-regulated circuits encompassing intricate networks mediated by neurotransmitters (e.g. acetylcholine, norepinephrine), neuropeptides (e.g. substance P, CGRP), immune cells (e.g. ILC2s, macrophages, T cells), and cytokines (e.g. TNF-α, IL-1β).

**Conclusions:**

Decoding key molecular targets within the neuroimmune axis provides novel strategies for predicting disease biomarkers (e.g. CGRP in early allergic inflammation) and refining therapeutic interventions (e.g. purposing anti-NGF biologics for neuropathic airway inflammation). This review specifically identifies promising targets – such as CGRP signalling in asthma and infections, and cholinergic pathways in COPD and lung cancer – for next-generation biologics and neuromodulatory therapies. This synthesis bridges neuroimmunology and clinical pulmonology, positioning neuroimmune crosstalk as a promising frontier in precision respiratory medicine and paving the way for an innovative therapeutic paradigm.

## KEY MESSAGES

Proposes a systematic framework for the pulmonary neuroimmune circuits, emphasizing its translational potential in biomarker discovery and therapeutic optimization.

Systematically examines emerging targets within neuroimmune crosstalk across five respiratory diseases (pulmonary infections, asthma, COPD, pulmonary fibrosis, lung cancer), encompassing neurotransmitters (e.g. acetylcholine), neuropeptides (e.g. CGRP), immunomodulatory cytokines (e.g. TNF-α), and their associated feedback regulatory circuits.

Bridges neuroimmunology and clinical pulmonology to advance the development of targeted precision therapeutic paradigms.

## 1. Introduction

To address ongoing environmental challenges, including pathogens and pollutants, organisms depend on specialized systems to detect threats, initiate rapid responses and restore homeostasis. Notably, the nervous and immune systems function as key sentinels in coordinating host defence mechanisms. Neurons detect danger through nociceptors and mechanoreceptors, subsequently releasing signals such as neurotransmitters to modulate effector responses for host protection [1]. Concurrently, immune cells identify molecular patterns indicative of damage or infection, triggering innate and adaptive immune responses, clearing damaged tissue, and facilitating tissue repair [2]. Interestingly, a ‘dialogue’ exists between neurons and immune cells, mediated by neurogenic factors and immune molecules, which allows them to respond reciprocally to each other’s signals. This interaction is exemplified by the presence of neurotransmitter receptors on immune cells and cytokine receptors on neurons [3], effectively integrating the nervous and immune systems into a cohesive network. Research indicates that neurotransmitters, such as norepinephrine (NE) and substance P (SP), play a role in modulating immune cell function, while cytokines, including tumour necrosis factor (TNF) and interleukin-6 (IL-6), communicate with neurons to regulate pain perception [4,5]. This bidirectional communication has led to the emergence of the transformative field of neuroimmunology, with seminal studies elucidating how neural circuits influence immune responses in contexts such as inflammation, injury and autoimmune diseases affecting the nervous system [6–9]. Notably, neural signals can elicit rapid reflexes on the millisecond scale, such as coughing, whereas immune responses typically unfold over minutes to hours; the interaction between these systems facilitates an optimized host defence.

The functional collaboration between the nervous and immune systems is established from the point of embryonic origin. In adult mammals, immune cells are derived from haematopoietic stem cells, with haematopoiesis predominantly occurring in the bone marrow. The autonomic innervation of the bone marrow is capable of regulating the development of both lymphoid and myeloid cell lineages [10]. Sympathetic nerves and stromal cells establish neuro-reticular complexes within the haematopoietic niche, which are instrumental in both the maintenance and differentiation of haematopoietic stem cells [11]. Research suggests that neuropeptides released by sensory nerve endings in the bone marrow positively influence haematopoietic stem cell activity and facilitate haematopoiesis. Additionally, ligands from the glial cell line-derived neurotrophic factor family are involved in the regulation of haematopoiesis [12]. This neuroimmune interaction, which is integral to both developmental processes and the maintenance of homeostasis, plays a significant role in the pathophysiology of various diseases, including inflammatory conditions affecting the skin, respiratory tract and gastrointestinal tract. These conditions are frequently associated with pain symptoms and compromised barrier function.

Among these barrier organs, the respiratory tract presents a compelling model for neuroimmune research. Its direct exposure to a wide range of environmental challenges – such as toxic chemicals, temperature variations, mechanical injuries and microbial pathogens – alongside its dense innervation and immunologically active mucosa, establishes it as a highly dynamic neuroimmune environment [13]. Anatomically, the lungs are characterized by an intricate network of neuroimmune interactions, wherein terminals of visceral sensory, sympathetic and parasympathetic nerves release neurotransmitters, such as acetylcholine (ACh), NE and calcitonin gene-related peptide (CGRP), in a paracrine manner. These neurotransmitters act on receptors located on adjacent alveolar macrophages, mast cells and dendritic cells, collectively modulating immune responses [14]. This structural proximity facilitates real-time bidirectional communication. For example, CGRP derived from nociceptors enhances type 2 innate lymphoid cell (ILC2) responses in asthma [15], whereas chronic allergen exposure can lead to structural and functional neuronal alterations, exacerbating symptoms such as cough and bronchospasm [16]. In chronic obstructive pulmonary disease (COPD), persistent inflammation stimulates the release of ACh from parasympathetic nerves, which in turn suppresses the secretion of pro-inflammatory cytokines by macrophages [17]. Given the extensive neuroimmune crosstalk in the lungs, its dysregulation is increasingly implicated in the pathophysiology of major respiratory conditions – diseases that pose substantial global public health challenges. According to data from the Global Burden of Disease Study 2021, asthma affects approximately 260 million individuals worldwide, ranking among the most prevalent chronic diseases and contributing significantly to global morbidity and mortality [18]. COPD is identified as the fourth leading cause of death globally, accounting for 3.5 million deaths in 2021 [19]. Additionally, the in-hospital mortality rate for patients with idiopathic pulmonary fibrosis (IPF) is approximately 50% [20]. Lung cancer remains the leading cause of cancer-related mortality globally, with nearly 2.5 million new cases and over 1.8 million deaths reported in 2022 [21]. This significant unmet medical need, against the backdrop of emerging evidence for pulmonary neuroimmunology, highlights the urgency of investigating neuroimmune hubs as novel therapeutic targets and underscores their therapeutic promise. Promising directions include investigating the potential of CGRP blockade in treating refractory asthma or modulating ACh to intervene in lung cancer progression.

Despite notable advancements in this field, there remains a paucity of comprehensive reviews on lung-specific neuroimmune circuits, their principal molecular mechanisms, and their roles in major respiratory diseases. In particular, Notably, the thorough investigation of the characteristic of ‘bidirectionality’ is insufficiently addressed, overlooking immune system-dominated pathways that modulate neural signals, such as immune-mediated neural sensitization and plasticity alterations. Consequently, this review concentrates on the concept of ‘bidirectional neuroimmune crosstalk’, scrutinizing the interactive framework between the pulmonary innervation network and resident immune cells to elucidate the distinctive neuroimmune microenvironment within the lungs. Building upon this foundation, we explore the bidirectional molecular pathway mechanisms underlying neuroimmune crosstalk in five major respiratory conditions – pulmonary infections, asthma, COPD, PF and lung cancer – selected for their significant global burden and the compelling, distinct evidence of neuroimmune dysregulation in each. We highlight their translational potential while also critically evaluating the limitations of current research and proposing future directions informed by emerging technologies. We anticipate that this work will offer innovative research perspectives for the field, facilitate the development of novel therapeutic strategies targeting the pulmonary neuroimmune axis, and ultimately foster breakthroughs in the precise diagnosis and treatment of respiratory diseases.

This narrative review is based on a comprehensive analysis of the literature available up to June 2025. A systematic search was performed across PubMed, Web of Science and Scopus databases. The search strategy combined free-text keywords with Medical Subject Headings (MeSH) in PubMed. Key terms were strategically combined using Boolean operators (AND, OR) to capture core concepts, including ‘pulmonary’, ‘lung’, ‘airway’, ‘neuroimmune’, ‘neurogenic inflammation’, ‘innervation’, and specific mediators (e.g. ‘Calcitonin Gene-Related Peptide’, ‘Substance P’, ‘acetylcholine’) and major respiratory diseases (e.g. ‘pulmonary infection’, ‘asthma’, ‘chronic obstructive pulmonary disease’, ‘pulmonary fibrosis’, ‘lung cancer’). Reference lists of retrieved articles were manually screened to identify additional foundational publications. Study selection was guided by their contribution to elucidating mechanistic insights or translational evidence of bidirectional neuroimmune crosstalk. Priority was given to high-impact original research and seminal reviews from the past decade to present a contemporary and evidence-based perspective on the field.

## 2. The lung and the neuronal network

During embryonic development, the formation of the respiratory tract is intricately associated with the establishment of an extensive neuronal network [22]. The human respiratory tract originates from the ventral foregut endoderm, and as the respiratory tree develops, a complex neural network emerges. This network is subject to dual neural regulation from both extrinsic and intrinsic neurons. Extrinsic neurons originate from the dorsal and ventral respiratory nuclei in the medulla oblongata, as well as the jugular and nodose ganglia [23]. These neurons have cell bodies located outside the respiratory tract, with axons that transmit signals *via* the vagus nerve to mediate parasympathetic efferent functions and most sensory afferent pathways [24]. In contrast, intrinsic neurons possess cell bodies distributed within the trachea and main bronchi, forming local ganglia. These neurons primarily receive signals from extrinsic neurons and relay them to target tissues throughout the respiratory tract.

The intricate pulmonary neuronal network is composed of neurons and glial cells located outside the central nervous system (CNS), typically organized into ganglia. Neurons innervating the lungs can be broadly categorized into autonomic neurons (including sympathetic and parasympathetic efferent neurons) and sensory afferent neurons. The sensory nerve terminals of vagal afferents are equipped with a variety of receptors, such as nociceptors, cough receptors and stretch receptors. These receptors play a critical role in detecting mechanical and chemical stimuli, integrating signals with autonomic neurons to facilitate essential physiological functions, including respiratory control and defensive reflexes (e.g. coughing), while also transmitting information regarding the pulmonary environment to the CNS. Parasympathetic efferent neurons convey signals from the brain, releasing neurotransmitters that interact with receptors located on bronchial smooth muscle, glands and the walls of blood vessels. This interaction results in bronchoconstriction, glandular secretion, vasodilation and mucosal swelling, thereby regulating pulmonary physiological functions. In contrast, sympathetic efferent fibres terminate at various points along the trachea and bronchi, releasing mediators such as adrenaline upon activation. This activation elicits physiological responses that oppose those of the parasympathetic system, including bronchodilation, inhibition of glandular secretion, vasoconstriction and a reduction in mucosal oedema. In the lungs, glial cells are predominantly non-myelinating Schwann cells, which envelop major airways and blood vessels and maintain close interactions with both parasympathetic and sympathetic neurons [25]. Glial cells have been primarily studied within the CNS and the gastrointestinal tract, where they are known to regulate neuronal homeostasis and neurotransmission, in addition to serving as immunologically active cells [26]. Although research into the specific roles of glial cells in the lungs is still in its nascent stages, studies have shown that these cells can secrete various neuroactive substances to modulate neuronal activity and detect local inflammation or injury signals, thereby contributing to the regulation of pulmonary immune responses [27]. Furthermore, glial cells facilitate the repair and regeneration of damaged neurons [28], playing a pivotal role in the pathogenesis and progression of lung diseases. Recent studies have reported that exosomal miR-21-5p derived from Schwann cells regulates tumour-associated immune suppression and evasion through the mitogen-activated protein kinase (MAPK), phosphatidylinositol 3-kinase (PI3K)/protein kinase B (Akt), and TNF signalling pathways, thereby promoting the proliferation, activation and metastasis of human lung cancer cells [29,30]. In a mouse model of pulmonary inflammation, non-myelinating Schwann cells upregulated the expression of chemokines C-X-C motif chemokine ligand 1 (CXCL1), CXCL2, and monocyte chemoattractant protein-1, and the cytokine leukaemia inhibitory factor, with their pro-inflammatory activity persisting for several hours post-treatment. This implies that glial cells might contribute to promoting and sustaining innate immune responses during lung infections [31]. Further research is necessary to clarify the exact mechanisms by which glial cells could serve as therapeutic targets in lung diseases, potentially aiding in the repair of inflammatory damage, maintaining lung health and preventing or treating lung cancer.

## 3. The lungs and the immune system

The respiratory mucosa plays a crucial role in preserving the integrity of the epithelial barrier, thereby providing defence against bacteria, pathogens and foreign antigens. It is also rich in lymphoid tissue and immune cells. The lung’s innate immune system includes neutrophils, macrophages, microglia, dendritic cells, mast cells and natural killer cells [32]. These cells identify pathogens and tissue damage through highly conserved pathogen-associated molecular patterns (PAMPs) and damage-associated molecular patterns (DAMPs), subsequently releasing cytokines and chemokines to initiate an inflammatory response. The adaptive immune system encompasses T cell-mediated antigen-specific cellular immunity and B cell-mediated humoral immunity, which together confer long-lasting immune protection. Innate lymphoid cells (ILCs) are a subset of innate immune cells that lack specific antigen recognition receptors but are essential for lymphoid organ development, tissue repair and defence against microbial infections. Dysregulation of ILCs is strongly linked to various diseases, including allergic conditions, chronic infections, metabolic disorders and tumours. Their functional characteristics are analogous to those of T lymphocytes in the adaptive immune system [33]. ILCs are categorized into distinct subgroups based on their surface markers, transcription factors, secreted cytokines and immunoregulatory functions. Specifically, ILC1 and ILC3 are involved in mediating immune responses against viral, intracellular bacterial and parasitic infections, whereas group 2 innate lymphoid cells (ILC2s) primarily facilitate allergic inflammation and tissue repair. Predominantly located in mucosal tissues, ILCs play crucial roles in maintaining tissue immune homeostasis, combating pathogenic infections, and augmenting T cell and B cell-mediated adaptive immune responses. Dendritic cells (DCs) are recognized as the most potent professional antigen-presenting cells within the body. They express a wide array of antigen-presenting molecules (major histocompatibility complex-I/II), co-stimulatory factors (CD80/B7-1, CD86/B7-2, CD40, CD40L), and adhesion molecules (intercellular cell adhesion molecule (ICAM)-1, ICAM-2, ICAM-3, lymphocyte function-associated antigen (LFA)-1, LFA-3), which facilitate efficient antigen capture, processing and presentation [34]. DCs are typically sparsely distributed across barrier mucosal sites that interface with the external environment, including the skin (as Langerhans cells), nasal cavity, lungs and gastrointestinal tract. Immature DCs possess a significant migratory capacity; upon activation, they migrate to lymphoid tissues where they interact with T cells and B cells to orchestrate and regulate immune responses. In contrast, mature DCs exhibit a high proficiency in activating naïve T cells, thereby serving as crucial mediators in the initiation, regulation and perpetuation of immune responses.

## 4. Neuroimmune communication in the lung

**Figure 1.**
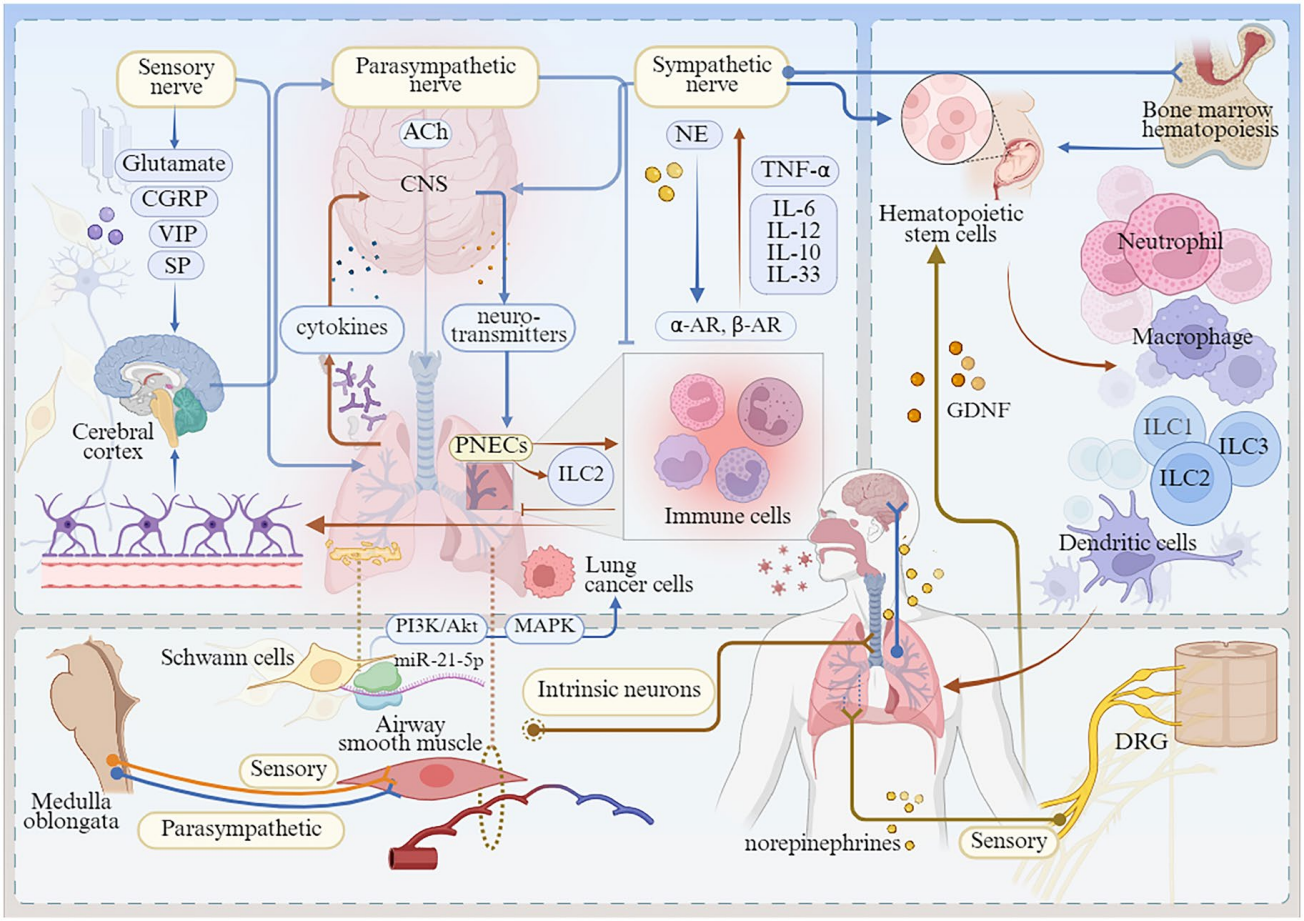
Pulmonary neuroimmune crosstalk landscape. Sensory afferent neurons, parasympathetic and sympathetic efferent neurons, and PNECs orchestrate immune responses in the lung. Local immune cells within the lung influence both peripheral neurons and the central nervous system. A variety of neurotransmitters, neuropeptides, and immune cytokines mediate neuroimmune interactions within the pulmonary environment. *Abbreviation:* CGRP, calcitonin gene-related peptide; VIP, vasoactive intestinal peptide; SP, substance P; NTS, neurotransmitters; ACh, acetylcholine; CNS, central nervous system; GABA, γ-aminobutyric acid; PNEC, pulmonary neuroendocrine cell; ILC2, group 2 innate lymphoid cell; α-AR, α-adrenergic receptors; β-AR, β-adrenergic receptor; HSC, haematopoietic stem cell; GDNF, glial cell line-derived neurotrophic factor; ILC1, group 1 innate lymphoid cell; ILC3, group 3 innate lymphoid cell; DC, dendritic cell; DRG, dorsal root ganglia; ASM, airway smooth muscle; NEBs, neuroendocrine bodies; SCs, Schwann cells; CA, cancer. (Created in BioRender (2025). https://BioRender.com/qc6qils).

A growing body of experimental models and *in vitro* studies demonstrates a close interplay between the nervous and immune systems in the regulation of lung function. Sensory afferent and autonomic efferent neurons interact with immune cells at distinct anatomical sites to form neuroimmune cell units [35]. The reciprocal interaction between the neural and immune systems involves a multitude of signalling molecules, including neuropeptides, neurotransmitters, cytokines and other molecular mediators, which are essential for communication and functional coordination between neurons and immune cells. Peripheral sensory neurons modulate immune responses by directly detecting tissue injury or pathogen presence, or by receiving signals such as chemokines and cytokines released from immune cells [36]. Simultaneously, immune cells themselves generate neuromodulatory mediators, collectively forming a dynamic bidirectional regulatory network between the two systems (Figure 1).

### 4.1. Neuromodulation of pulmonary immunity

Generally, the lungs are innervated by three primary types of nerve fibres: sensory afferent fibres, parasympathetic efferent fibres and sympathetic efferent fibres. Each type of fibre plays a crucial role in modulating the function and activity of pulmonary immune cells through distinct pathways. Notably, vagal afferent fibres innervating the lungs serve as key mediators of neuroimmune interactions [37].

#### 4.1.1. Regulation of the pulmonary immune microenvironment by peripheral nociceptors

Nociceptors, located at the peripheral terminals of primary sensory neurons, are extensively distributed in barrier tissues and pulmonary organs. These nociceptors and visceral afferent nerves release a variety of neuropeptides and neurotransmitters from their peripheral terminals, including CGRP, SP, vasoactive intestinal peptide (VIP), ACh, γ-aminobutyric acid (GABA), NE, adenosine triphosphate and l-glutamate. These mediators significantly influence the functions of both innate and adaptive immune cells. DCs, neutrophils, macrophages, mast cells and T cells within lung tissue express receptors for these neuronal mediators, facilitating direct responses to nociceptor activation and the initiation of ‘neurogenic inflammation’ [22]. This response is characterized by nociceptor sensitization, endothelium-dependent vasodilation, mast cell degranulation, oedema, local activation of DCs, macrophages and T cells, as well as immune cell extravasation. For example, SP, through its interaction with neurokinin-1 receptors, enhances the survival of activated T cells [38], stimulates the production of pro-inflammatory mediators by macrophages and promotes neutrophil chemotaxis and migration [39]. Conversely, CGRP acts as a negative regulator of innate immune responses, serving a protective role in mitigating tissue damage during inflammatory diseases. CGRP inhibits the production of inflammatory cytokines by macrophages and DCs and reduces antigen presentation to T cells by upregulating IL-10 expression and suppressing nuclear factor-kappa B activity. These effects are mediated through downstream pathways involving cyclic AMP-protein kinase, which is activated by the CGRP receptor [40]. Additionally, CGRP reduces monocyte recruitment, diminishes the macrophage-killing capacity and suppresses ILC2 responses [41]. In a murine model of lipopolysaccharide-induced acute lung injury, CGRP attenuates inflammation by modulating macrophage polarization, reducing the activation of the NOD-like receptor family pyrin domain containing 3 (NLRP3) inflammasome and pro-IL-1β mRNA expression, while enhancing IL-4-induced M2 macrophage markers, such as IL-10[42].

The primary excitatory neurotransmitter of sensory afferent neurons is L-glutamate. Glutamate exacerbates airway inflammation by promoting the secretion of pro-inflammatory cytokines *via* N-methyl-D-aspartate receptors, thereby contributing to acute lung injury and various inflammatory lung conditions, including pulmonary oedema, fibrosis and lung cancer [43]. Furthermore, vagal afferents are capable of initiating sensory-autonomic neuroimmune reflexes, such as the ‘cholinergic anti-inflammatory reflex’ [44]. Inflammatory signals within the body are transmitted to vagal efferent fibres *via* reflex pathways, wherein these efferent signals exert anti-inflammatory effects across various inflammatory diseases by targeting immune cells, such as T cells and macrophages, which express choline acetyltransferase. This process involves the downregulation of tumour necrosis factor-alpha (TNF-α) production by peripheral macrophages.

#### 4.1.2. Autonomic efferent neural regulation of pulmonary immune responses

Both the sympathetic and parasympathetic efferent nervous systems are integral in modulating pulmonary immune responses. The sympathetic nervous system generally provides broad and coordinated regulation of immune responses, affecting the function, survival, proliferation, circulation and trafficking of immune cells. In contrast, the parasympathetic nervous system typically functions within individual organs, responding to local signals [36].

Sympathetic outflow originates from preganglionic neurons located in the intermediolateral cell column of the thoracolumbar spinal cord, with NE serving as its principal neurotransmitter [36]. NE plays a crucial role in antigen processing and lymphocyte activation [45], thereby facilitating their entry into circulation. Several *in vitro* studies have demonstrated that sympathetic nervous system output modulates DCs, T cells and B cells through β2-adrenergic receptor (β2AR) signalling pathways. Activation of β2AR on DCs inhibits the cross-presentation of antigens to CD8+ T cells and reduces the secretion of pro-inflammatory cytokines such as TNF-α, IL-12, and IL-6, while enhancing the production of anti-inflammatory cytokines IL-10 and IL-33. Furthermore, β2AR activation promotes responses from Th2 and regulatory T cells, while suppressing Th1 cell and ILC2 responses, and diminishing the number and activity of circulating natural killer cells [46,47]. Activation of α1-adrenergic receptors (α1AR) facilitates the migration of dendritic cells to lymph nodes, whereas α2AR enhances antigen uptake by DCs. Beyond NE, certain sympathetic efferent neurons release neuropeptide Y (NPY) and other signalling molecules that may influence immune cell function.

Parasympathetic efferent innervation originates from local and intramural ganglia, receiving excitatory cholinergic inputs from preganglionic neurons located in the brainstem and sacral spinal cord [36]. The parasympathetic nervous system predominantly releases the neurotransmitter ACh, which facilitates the contraction of airway smooth muscle and promotes mucus gland secretion through the activation of M3 and M1 muscarinic receptors. Conversely, M2 muscarinic receptors provide negative feedback regulation to mitigate excessive ACh release [48]. Additionally, cholinergic signalling plays a crucial role in modulating immune responses *via* muscarinic receptors, contributing to what is termed the ‘cholinergic anti-inflammatory pathway’, particularly within the pulmonary system, where it is referred to as the ‘pulmonary parasympathetic inflammatory reflex’ [49]. During pulmonary infections, this reflex interacts with the spleen – a vital component of the cholinergic anti-inflammatory pathway – to regulate the recruitment of α7 nicotinic acetylcholine receptor-positive CD11b-positive cells from the spleen to inflamed lung tissue. This recruitment is instrumental in diminishing circulating pro-inflammatory cytokine levels, thereby locally attenuating the severity of infection and inflammation in the lungs. Furthermore, cholinergic neurons influence the expression of α7 nicotinic acetylcholine receptor (α7nAChR) on ILC2s, and the activation of α7nAChR on ILC2s suppresses the production of innate cytokines, which in turn reduces airway hyperreactivity [50]. Vagal cholinergic input activates pulmonary group 3 innate lymphoid cells (ILC3s) to produce IL-22, a critical mediator of mucosal immunity. Furthermore, parasympathetic nerves may release non-cholinergic neurotransmitters, such as nitric oxide and VIP, which facilitate bronchial smooth muscle relaxation while inhibiting mucus secretion and inflammatory responses [36].

#### 4.1.3. PNECs in modulating pulmonary immune responses

In addition to the conventional free nerve endings of nociceptive neurons in peripheral tissues, specialized structures also fulfil a neuroendocrine role in immune regulation. Within the lung, pulmonary neuroendocrine cells (PNECs), a subset of endoderm-derived cells, are integral components of the airway epithelium, coexisting with ciliated, goblet, basal and brush cells. PNECs receive innervation from sensory afferent nerves originating in the nodose and dorsal root ganglia, as well as cholinergic efferent nerves from the brainstem and intrinsic ganglia. Positioned in proximity to pulmonary ILC2s and peptidergic neurons [51], PNECs secrete GABA and CGRP under the regulation of neurotrophic factor 4-dependent neural circuits established during development [52]. Notably, PNECs regulate goblet cell hyperplasia *via* GABA and modulate ILC2 activity through the production of CGRP. Recent studies have shown that CGRP acts as an environmentally dependent negative regulator, modulating ILC2 responses to alarmins and neuropeptides [41,53]. Specifically, CGRP activates the MAPK pathway, which induces keratinocyte proliferation and the production of TNF-α, IL-1β, IL-6, and nerve growth factor (NGF). Furthermore, CGRP and SP enhance NLRP1/caspase-1 inflammasome signalling in keratinocytes, leading to IL-1β-dependent mechanical hyperalgesia. In the context of pulmonary diseases, dysfunction of PNECs results in excessive CGRP release, thereby contributing to heightened immune cell recruitment [51].

### 4.2. Immunoregulatory role of pulmonary neurobiology

It is well-established that resident immune cells in the lung integrate neuron-derived signals to maintain tissue homeostasis and facilitate defence mechanisms. Conversely, immune cells are capable of synthesizing and releasing neuromodulators such as ACh, dopamine, NE, enkephalins, endorphins and various inflammatory mediators. These substances further modulate neuronal and immune responses, contributing to the formation of complex neuroimmune regulatory circuits [54]. Neuronal cell bodies and their terminals express receptors that are responsive to cytokines, lipids, growth factors and proteases originating from immune cells, thereby influencing neuronal responses. Peripheral nociceptor nerve endings are equipped with specific receptors and ion channels that detect and respond to various molecular mediators released by immune cells, playing a pivotal role in the generation and modulation of pain. Upon activation, action potentials are transmitted to the nociceptor cell bodies located in the dorsal root ganglia (DRG) and subsequently relayed to the spinal cord and brain, where they are interpreted as pain. During inflammatory conditions, the threshold for nociceptor activation is lowered, resulting in pain hypersensitivity or neuronal sensitization. This increased perception of pain is closely associated with tissue injury and the subsequent cascade of inflammatory responses.

#### 4.2.1. Modulation of the peripheral nervous system by pulmonary immune cells

Pathogens invading the respiratory system, along with their associated inflammatory mediators, can modulate the activity of local sensory neurons. Pathogens or damage to lung tissue induce immune cells to release inflammatory mediators, including pro-inflammatory cytokines such as TNF-α, IL-1β, IL-6, IL-17, and IL-5, as well as NGF, prostaglandin E2, serotonin and histamine [55]. Cytokines and other inflammatory mediators interact with receptors located on the peripheral axon terminals of sensory neurons, including cytokine receptors, G protein-coupled receptors and type 1 tyrosine kinase receptors. Beyond immune activation, pathogens can directly activate sensory neurons by engaging PAMPs with pattern recognition receptors (PRRs) on neurons, such as toll-like receptor 4 (TLR4) [56].

Furthermore, research has demonstrated that immune cells within the lung modulate neuronal activity through various pathways. DCs, upon stimulation by pathogens, release GABA, which acts on local neurons to modulate the inflammatory response, thereby reducing tissue damage and decelerating pathological progression [57]. Nie et al. [58] reported that eosinophils in the lung facilitate bronchoconstriction and airway hyperresponsiveness (AHR) by amplifying vagal reflexes. In the lung, eosinophils not only increase the density of sensory nerves in the airway epithelium [59] but also adhere to airway epithelial nerves *via* intercellular adhesion molecule-1 [60], thereby influencing vagally-induced bronchoconstriction. During allergic reactions in the pulmonary system, mast cells release neurotransmitters such as histamine, serotonin, and SP, which directly interact with bronchial nerve terminals, resulting in bronchospasm, increased mucus secretion, and enhanced inflammatory responses [61].

#### 4.2.2. Influence of peripheral immune cells on the CNS

Peripheral neuroimmune interactions within lung tissue can exert an influence on the CNS. Nociceptive neurons express and release various mediators, including CGRP, chemokine (C-C motif) ligand 2 (CCL2), chemokine (C-X3-C motif) ligand 1 (CX3CL1), and TNF-α, into the spinal cord *via* their central nerve terminals. These mediators are crucial in activating microglia, the primary immune cells involved in the central sensitization mechanisms underlying pain [62]. Microglia, as the resident innate immune cells of the CNS, are capable of producing pro-inflammatory factors, such as TNF-α and IL-1β, as well as neurotrophic factors, including brain-derived neurotrophic factor (BDNF) [63]. When peripheral afferent nociceptive neurons release CX3CL1 into the spinal cord, it activates microglia to produce TNF-α. Subsequently, TNF-α stimulates spinal astrocytes, leading to the production of CCL2. Through its receptor CCR2, CCL2 activates central neurons, thereby contributing to the development of neuropathic pain [64]. In chronic pain conditions, T cells infiltrate the spinal cord and contribute to neuronal sensitization. Consequently, the interaction between peripheral nociceptive neurons, spinal microglia and astrocytes facilitates the central sensitization of neuronal circuits [54], forming a complex neuroimmune network that connects the lungs to the CNS. Moreover, research by Francesca Odoardi and colleagues [65] has demonstrated that the lungs serve as a niche for the long-term survival and maturation of T cells involved in multiple sclerosis, a T cell-mediated autoimmune disease of the CNS. During episodes of local lung inflammation, autoreactive T cells become activated, undergo extensive proliferation and subsequently migrate into the CNS, triggering autoimmune pathology. This process may represent a mechanism by which pulmonary inflammatory responses contribute to the onset of multiple sclerosis.

### 4.3. Neuroimmune interactions in the lung

In the lungs, neurons and immune cells are in close proximity, enabling direct interactions. Previous research has predominantly focused on the unidirectional regulation of immune cells by the nervous system, emphasizing the primary role of neural control. Neurons secrete neurotransmitters and neuropeptides that influence pulmonary vasculature, lymphatic function and the polarization of innate and adaptive immune responses. Nonetheless, with the burgeoning interest in neural-immune interactions in recent years, accumulating evidence supports the concept that immune cells can actively modulate neuronal activity in diverse ways. Immune cells are capable of forming intricate feedback loops with neural regulatory pathways, establishing both positive and negative feedback circuits along the neuroimmune axis. For example, immune cells release a variety of mediators, including lipids, cytokines and growth factors, which act on peripheral tissues and the spinal cord, thereby contributing to neuronal plasticity and chronic pain [66]. Immune cells play a pivotal role in sensitizing nociceptive sensory neurons and influence neuronal receptor gene expression, neuronal morphological remodelling, and the modulation of neurotransmitter synthesis and release [67].

Immune cells are not merely targets of sensory afferent and autonomic efferent signals but also function as regulators of afferent neural signalling. Various immune cell types produce neurotransmitters and neuroregulatory peptides, including NE, ACh, SP, CGRP and VIP [68]. For example, T lymphocytes are capable of synthesizing and regulating the production of catecholamines and ACh. These neurotransmitters subsequently exert paracrine effects that modulate immune responses, thereby influencing the production of various cytokines, including ILs, members of the TNF family, chemokines and their receptors.

Recent research has started to clarify the cellular and molecular components involved in bidirectional neural-immune interactions. Accumulating evidence suggests that environmental signals are perceived by distinct neuro-immune cellular units (NICUs), which are specific anatomical sites where immune cells and neurons co-localize and interact functionally. NICUs are increasingly recognized as pivotal coordinators of various physiological and pathological processes [35]. A prominent example is the ‘lung sensory neuron–ILC2’ unit, a representative pulmonary neuro-immune cellular unit. ILC2s receive signals from epithelial cells, neurons and other immune cells, subsequently regulating downstream effector cell functions and playing critical roles in barrier tissues. Notably, ILC2s express VIP receptor 2, and activation of this receptor induces IL-5 secretion, thereby promoting eosinophil recruitment [69]. In turn, IL-5 signals back to pulmonary nociceptors, promoting the release of VIP and further activating ILC2s [70]. It is noteworthy that this positive feedback loop can be disrupted through the pharmacological silencing of pulmonary sensory neurons, indicating that the modulation of the ‘pulmonary sensory neuron-ILC2’ interaction may offer a promising therapeutic approach for allergic diseases. Moreover, research has shown that the pulmonary cholinergic neuron-ILC2 axis can initiate tissue protective mechanisms *via* rapid neuroimmune signalling. Specifically, pulmonary cholinergic neurons release neuropeptide U (NMU), which activates ILC2s, leading to the secretion of IL-5 and IL-13. This cascade results in an increase in pulmonary mast cells and eosinophils, thereby enhancing anti-helminth responses within the lungs [71]. Additionally, IL-13 has been demonstrated to upregulate NMU expression in dorsal root ganglion neurons, suggesting the existence of NMU-expressing neurons within the pulmonary cholinergic neuron-ILC2 – IL-13 regulatory loop. This finding has significant implications for the targeted modulation of host ILC2 responses. In summary, the multicellular frameworks of NICUs present significant challenges to the prevailing understanding of tissue physiology and pathology coordination. NICUs are poised to become one of the most promising areas of exploration in immunology over the coming decades. In-depth investigations into the cellular and molecular characteristics of NICUs have the potential to uncover novel therapeutic targets that remain underexplored (Table 1).

**Table 1.** Elements involved in pulmonary neuroimmune communication.

Nerve/neuron	Neural signals	Immune cells	Immune molecules/cytokines	Refs
Nociceptor neurons	SP	T cells, macrophages, neutrophils	TNF-α, IL-1β, IL-6	[38,39]
Nociceptor neurons	CGRP	Macrophages, dendritic cells, T cells; Monocytes, macrophages; Macrophages	IL-10; IL5, IL-13; NLRP3, IL-1β, IL-10	[40–42]
Sensory afferent neurons	l-glutamate	Macrophages, dendritic cells, T cells, B cells	–	[43]
vagal afferent	ACh	T cells, macrophages	TNF-α	[44]
Sympathetic nerve	NE	Dendritic cells, Th2 cells, Tregs, B cells, NK cells	TNF-α, IL-12, IL-6, IL-10, IL-33	[45–47]
Cholinergic neurons	ACh	ILC2s; ILC3s	IL-4, IL-5, IL-10, IL-13; IL-22	[49,50]
PNEC	GABA, CGRP	ILC2s	TNFα, IL-1β, IL-6	[51–53]
Peripheral neurons	GABA	Dendritic cells	–	[57]
Vagus nerve	ACh	Eosinophils	IL-5	[58–60]
Sensory neurons	Histamine, serotonin, SP	Mast cells	–	[61]
Nociceptor neurons	CGRP, BDNF	Microglia	TNFα, IL-1β	[62–64]
Nociceptor neurons	VIP	ILC2s, Eosinophils	IL-5	[69,70]
Cholinergic neurons	NMU	ILC2s, Mast cells, eosinophils	IL-5, IL-13	[71]

Principal cellular and molecular components mediating communication between the pulmonary nervous and immune systems, encompassing neurons, neural signals, immune cells and cytokines.

*Abbreviations:* SP, substance P; CGRP, calcitonin gene-related peptide; NLRP3, nucleotide-binding oligomerization domain-like receptor protein 3; ACh, acetylcholine; NE, Norepinephrine; PNEC, Pulmonary neuroendocrine cell; GABA, γ-aminobutyric acid; BDNF, brain-derived neurotrophic factor; VIP, vasoactive intestinal peptide; NMU, neuromedin U.

## 5. Mechanism of neuroimmune crosstalk in lung disease

Both the pulmonary immune system and the nervous system have developed rapid and coordinated mechanisms to sense environmental changes and mount appropriate responses. Recent studies have revealed bidirectional communication between these systems in common respiratory diseases, mediated by cellular and molecular mechanisms involving soluble factors such as neurotransmitters, neuropeptides and immune cell cytokines that signal through cell surface receptors. This section examines the mechanisms of neuroimmune crosstalk in several common pulmonary diseases, with a particular focus on the bidirectional feedback loops involved, to elucidate the current research landscape (Table 2).

**Table 2.** Critical neuroimmune crosstalk and its translational potential in respiratory diseases.

Disease	Mechanisms of neuroimmune crosstalk	Potential clinical translation	Refs
Pulmonary Infections	Specific agonists for NE or β2AR are capable of inhibiting the expression of cytokines like TNFα, CCL2 and CXCL2 in alveolar macrophages, and simultaneously suppressing the expression of immunoregulatory factors IL-4, IL-5, and IL-13.	NE or β2AR agonists may help suppress inflammatory responses during pulmonary infections.	[56,74]
	CGRP inhibits the expression of immune cell chemokine CXCL1, hindering neutrophils from reaching the infection site; it prevents NLRP3 inflammasome activation and reduces the secretion of mature IL-1β.	CGRP antagonists may enhance immune responses and bacterial clearance in lung infections.	[75,76]
	Pneumonia activates GABAergic neurons in the brain, which subsequently drives pulmonary sympathetic nerves to release NE, activating local ADRB2^+^ macrophages in the lungs and thereby exacerbating cytokine storms and pulmonary injury.	Targeted inhibition of the brain GABAergic neuron-pulmonary sympathetic nerve-NE-ADRB2^+^ macrophage axis may represent a novel therapeutic strategy for severe pneumonia.	[196]
Asthma	Allergens stimulate PNECs to release neurotransmitters (serotonin and GABA) and neuropeptides (SP, Neurokinin A, VIP, CGRP), transmitting signals to ILC2s and regulating airway immune responses.	Targeted modulation of the neuroimmune interaction pathway of the PNEC-ILC2 axis holds promise as a therapeutic strategy for asthma control.	[16,87]
	Autonomic nerve fibres generate ACh and NPY to modulate ILC2s. NMU and VIP can boost ILC2 function, whereas CGRP, adrenaline and dopamine can restrain the ILC2 response in mucosal tissues.	Targeting key molecular components of the airway nerve fiber-ILC2 axis may improve immune responses in asthmatic patients.	[53,71,88]
	IL-5 exposure significantly increases sensory nerve density in the airway epithelium and eosinophil counts, elevating AHR.	Anti-IL-5 monoclonal antibodies selectively suppress eosinophilic inflammation and reduce acute exacerbations in severe asthma patients.	[192,193]
	CGRP suppresses ILC2-mediated allergic inflammation; proximally stimulates ILC2s and induces the generation of cytokines IL-5 and IL-13, triggering the immune response.	Precise modulation of local CGRP concentrations may ameliorate allergic asthma.	[41,89]
	SP can promote the chemotaxis of eosinophils and mast cells.	The development of SP antagonists is helpful for the treatment of asthma.	[14]
	The NMU/NMUR1 axis can stimulate ILC2s to produce rapid effects and potentially initiate eosinophil inflammatory reactions in the early phase.	NmU signal transduction antagonists hold great potential as therapeutic targets for treating allergic inflammation.	[91]
	GABA induces goblet cell proliferation and mucus secretion to cope with allergy.	The beneficial effects of GABA agonists on patients with asthma may be a feasible new treatment.	[87]
	Chronic allergen exposure can induce structural and functional changes in cholinergic neurons and enhance AHR.	Reducing the remodelling of cholinergic neurons may be a potential target for preventing and relieving asthma symptoms.	[92,93]
	Sympathetic neuron-derived dopamine inhibits the mitochondrial oxidative phosphorylation pathway of ILC2s, thereby suppressing allergic inflammation in the lungs.	Local dopamine administration may attenuate allergic inflammation and asthma pathogenesis.	[94,95]
	ATP released by neurons can activate various immune cells (including eosinophils, mast cells, dendritic cells and alveolar macrophages) and directly participate in airway inflammation; purinergic receptors (ADORA1) are also associated with asthma susceptibility.	Targeted suppression of purinergic signaling may serve as an effective therapeutic strategy for asthma.	[96–102]
COPD	The increases in MSNA and NE correlate with inflammatory responses in circulation and elevated levels of IL-6.	Regulation of sympathetic nerve activity may alleviate the inflammatory response of COPD.	[106]
	Chronic pulmonary inflammation stimulates parasympathetic neurons to release ACh, thereby activating α7nAChR on macrophages and neutrophils and alleviating pulmonary inflammation and injury.	Regulating the activation of α7nAChR on immune cells through ACh is beneficial for alleviating the pulmonary inflammatory injury caused by COPD.	[107,108,204]
	Airway inflammation and injury stimulate the terminals of the vagus nerve, promoting the activation of M1R and M3R by ACh, thereby inducing airway constriction and inflammatory responses.	The blocking of airway constriction and the anti-inflammatory effects of muscarinic antagonists may contribute to symptomatic improvement in patients with COPD.	[109,110,191]
	Bombesin secreted by PNEC induces abnormal airway remodelling and promotes the progression of COPD.	Targeted regulation of PNECs may be a therapeutic target for COPD.	[115]
	NGF activates TrkA and p75NTR and continuously participates in the progressive inflammatory process of COPD.	Local blockade of NGF receptor signaling may decelerate COPD progression.	[117,118]
	Elevated serum levels of VIP correlate with the acute exacerbation episodes in COPD; inhaled VIP treatment can limit proinflammatory cytokines (TNF-α, IL-1β and IL-6) in lung tissue, increase superoxide dismutase and relieve COPD symptoms by anti-inflammatory and anti-oxidation.	Rational use of VIP or its derivatives in the treatment of different stages of COPD.	[119–121]
PF	The deficiency of IFNγ signal transduction leads to the overactivation of ILC2 during the inflammatory phase and accelerates the progression of PF.	Activation of IFNγ signaling may decelerate PF progression.	[128,129]
	Catecholaminergic neurons secrete NE that binds to β2AR, capable of inhibiting the ILC2 response.	NE agonists may ameliorate inflammatory responses.	[130]
	NMU produced by cholinergic neurons can activate ILC2 and rapidly express the type 2 inflammatory cytokines (IL-5 and IL-13), aggravating PF.	NMU antagonists may help ameliorate PF.	[131,132]
	NGF promotes the activation and migration of pulmonary immune cells, intensifying the local inflammatory response and the degree of fibrosis in lung tissue. Conversely, inflammatory factors released by immune cells can stimulate the production of NGF, further accelerating the deterioration of PF.	NGF antagonists may represent a novel strategy to decelerate PF progression.	[133,134]
	The decreased levels of VIP lead to enhanced activity of NFAT, aggravating pulmonary inflammation and fibrosis.	VIP may be an effective therapeutic target for the treatment of PF	[135]
	VIP can reverse EMT by restoring autophagy in alveolar epithelial cells; downregulate IL-17RC in fibroblasts to block the activation of fibroblasts.		[136,137]
	NPY and the Y1R antagonists can inhibit the release of IL-1β and impede EMT of human alveolar epithelial cells.	Exogenous NPY and Y1R antagonists may be a potential therapeutic strategy to delay the progression of pulmonary fibrosis.	[138]
	SP binds to the NK1 receptor, activating alveolar macrophages and promoting the release of inflammatory mediators (TNF-α and IL-1β); in turn, the inflammatory mediators can stimulate nerve endings and increase SP release, exacerbating pulmonary inflammation and fibrosis.	Targeted suppression of SP/NK-1 receptor signaling may mitigate PF.	[139,140]
	Dopamine and NE released by the sympathetic nerves bind to specific receptors and then stimulate pulmonary macrophages to release inflammatory mediators such as TNF-α, IL-1β, IL-6, and IL-8; in turn, the inflammatory mediators can act on the sympathetic nerves in a reverse manner, enhancing the synthesis and secretory activity of neurotransmitters and accelerating the exacerbation of pulmonary inflammation and fibrotic reactions.	Dopamine or NE may constitute effective therapeutic targets for reducing pulmonary inflammatory injury and fibrosis.	[141,142]
	TNF-α inhibits the activity of vagus nerve, reduces the release of ACh and anti-inflammatory mediators and aggravates pulmonary fibrosis.	Inhibiting TNF-α while enhancing vagal nerve activity may confer protection against PF.	[143,144]
Lung Cancer	Knocking out TRPV1 expressed by sensory afferent neurons can inhibit GABA secretion in NSCLC, reduce immune cell infiltration and enhance anti-tumor immune responses; B cells can also synthesize and secrete GABA to regulate anti-tumor immune responses.	GABA agonists might improve lung cancer prognosis.	[152–155]
	ACh activates the EGFR/PI3K/AKT signaling pathway through M3R and upregulates the secretion of the pro-inflammatory factor IL-8, thereby promoting the proliferation, invasion and migration of NSCLC cells.	Down-regulating M3R, blocking the EGFR/PI3K/AKT pathway, and inhibiting IL-8 signaling may be important therapeutic interventions targeting the tumor microenvironment of NSCLC.	[160]
	ACh activates the WNT pathway through M3R and mediates the tolerance of NSCLC cells to chemotherapy drugs; immune cells in the tumor microenvironment (including T cells, B cells and macrophages) also synthesize and release ACh, exerting immunomodulatory functions.	Targeting the ACh/M3R/WNT axis represents a promising therapeutic strategy to help alleviate the degree of DTP cell formation in NSCLC and prevent tumor recurrence.	[163–165]
	NGF promotes the release of NE from nerves in the tumor microenvironment, which binds to α1-AR expressed on immune cells, thereby affecting the activation of immune cells and the production of inflammatory factors. The inflammatory factors TNF-α and IL-6 can inversely stimulate the production of NGF and promote tumor growth and metastasis.	The chemotherapeutic drugs targeting NGF may inhibit the growth of lung cancer.	[171–173]
	SP upregulates the expression of TLR-4 by binding to the NK1 receptor on tumor cells, thereby increasing the proliferation and invasion activities of tumor cells.	Reducing TLR-4 expression by regulating the SP/NK-1R pathway may be an effective supplement for the treatment of lung cancer.	[175,176]
	SCLC induces T cell-mediated immune responses by expressing neuron-specific antigens, prompting the body to produce various antibodies that may erroneously attack normal neuron cells in the CNS, resulting in paraneoplastic neurological autoimmune diseases. Meanwhile, a large number of cytokines (TNF-α, IL-6) released after immune system activation will further exacerbate the inflammatory response and nerve damage.	The combination of neuroprotective drugs and immunomodulatory therapy may effectively improve the paraneoplastic neurological disorders of lung cancer.	[179,180]

Summary of pivotal mechanisms and therapeutic implications of neuroimmune interactions across five major respiratory pathologies: pulmonary infections, asthma, chronic obstructive pulmonary disease (COPD), pulmonary fibrosis (PF), and lung cancer.

*Abbreviations:* NE, Norepinephrine; β2AR, β2-adrenergic receptor; TNFα, tumour necrosis factor; CCL2, chemokine (C-C motif) ligand 2; CXCL2,; IL, interleukin; CGRP, calcitonin gene-related peptide; NLRP3, nucleotide-binding oligomerization domain-like receptor protein 3; GABA, γ-aminobutyric acid; PNECs, Pulmonary neuroendocrine cells; SP, substance P; VIP, vasoactive intestinal peptide; ILC2s, group 2 innate lymphoid cells; ACh, acetylcholine; NPY, neuropeptide Y; NMU, neuromedin U; AHR, airway hyperresponsiveness; MSNA, muscle sympathetic nerve activity; M1R, muscarinic acetylcholine receptor M1; M3R, muscarinic acetylcholine receptor M3; IFNγ, interferon-γ; NGF, nerve growth factor; EMT, epithelial-mesenchymal transition; NK1, neurokinin-1 receptor; TRPV1, transient receptor potential vanilloid 1; EGFR, epidermal growth factor receptor; PI3K, phosphatidylinositol 3-kinase; AKT, protein kinase B; NSCLC, non-small cell lung cancer; TLR-4, toll-like receptor 4; SCLC, small cell lung cancer; CNS, central nervous system.

### 5.1. Pulmonary infections

**Figure 2.**
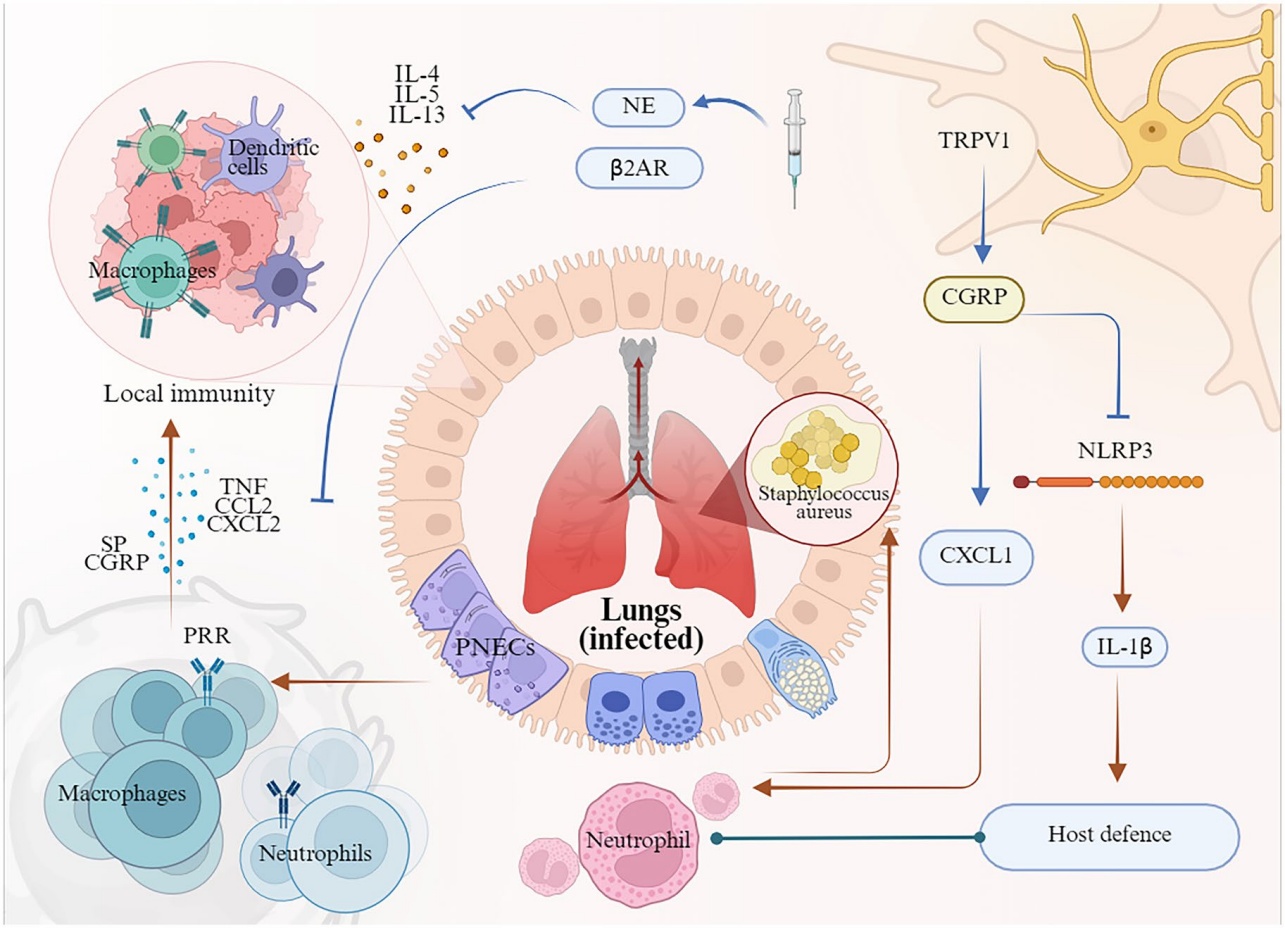
Neuroimmune interactions in pulmonary infections. Pulmonary local immunity and neural regulation collaboratively modulate host defence mechanisms against pulmonary pathogens. For instance, during a Staphylococcus aureus infection in the lungs, immune cells detect PAMPs/DAMPs via PRRs, thereby triggering innate immune responses. TRPV1 vagal neurons can release CGRP, which subsequently inhibits the expression of the chemokine CXCL1 and the activation of the NLRP3 inflammasome. This action serves to mitigate neutrophil and IL-1β-mediated host defence responses. *Abbreviations:* PNECs, pulmonary neuroendocrine cells; PRR, pattern recognition receptor; SP, substance P; CGRP, calcitonin gene-related peptide; TNF, tumour necrosis factor; CCL2, C-C motif chemokine ligand 2; CXCL2, C-X-C motif chemokine ligand 2; NE, norepinephrine; β2AR, β2-adrenergic receptor; TRPV1, Transient receptor potential vanilloid 1; CXCL1, C-X-C motif chemokine ligand 1; NLRP3, pyrin domain containing 3; IL-1β, interleukin-1β. (Created in BioRender (2025). https://BioRender.com/r84u013).

The lung, as a primary barrier surface exposed to the external environment, is particularly vulnerable to infections. Pulmonary infections and severe pneumonia continue to pose significant public health challenges, frequently resulting in mortality, particularly among children, immunocompromised individuals, and the elderly [72]. Staphylococcus aureus, a Gram-positive bacterial pathogen, is a predominant cause of nosocomial infections, especially those involving the respiratory tract, such as ventilator-associated pneumonia [73]. The emergence of methicillin-resistant *Staphylococcus aureus* (MRSA) strains underscores the necessity for developing non-antibiotic therapeutic strategies. Targeting neuroimmune signalling pathways represents a potential strategy to augment host immunity against pulmonary pathogens (Figure 2).

It is well-established that during pulmonary infections, immune cells recognize PAMPs or DAMPs through PRRs, such as Toll-like receptors and Nod-like receptors. These receptors identify pathogenic microorganisms and initiate the production of chemokines and inflammatory mediators, thereby triggering innate immune responses. Nociceptive sensory neurons, which express receptors for inflammatory mediators, detect danger signals conveyed by activated immune cells. In response, these neurons secrete cytokines, chemokines (e.g. TNF, IL-1α, IL-6, CCL2, and CXCL1), and neuropeptides (e.g. SP, CGRP, VIP, galanin and pituitary adenylate cyclase-activating polypeptide), thereby modulating local immune responses [56]. For instance, specific agonists of the sympathetic neurotransmitter NE or β2AR can downregulate the expression of cytokines and chemokines such as TNFα, CCL2, and CXCL2 in alveolar macrophages following lipopolysaccharide stimulation. These agonists also inhibit the expression of IL-4, IL-5, and IL-13 induced by IL-33, thus negatively regulating innate immune responses and mitigating inflammation during pulmonary infections [74]. In the context of MRSA lung infection, transient receptor potential vanilloid 1 (TRPV1)-expressing vagal nociceptors release the neuropeptide CGRP into the airways. CGRP suppresses the expression of the chemokine CXCL1, consequently impairing neutrophil recruitment to the site of infection in the lungs [75]. Additionally, CGRP inhibits the activation of the NLRP3 inflammasome and reduces the secretion of mature IL-1β [76], thereby inhibiting neutrophil- and IL-1-mediated host defences. Experimental investigations have shown that the selective ablation of TRPV1-expressing neurons, targeting the antagonism of CGRP, results in improved survival rates, enhanced neutrophil and γδ T cell responses and increased bacterial clearance in a murine model of MRSA-induced fatal pneumonia [75]. Consequently, CGRP emerges as a promising clinical target for pneumonia therapy. Importantly, mechanisms extending beyond CGRP signalling, such as those involving glutamate, ATP and other neuropeptides like SP, neurokinin A and VIP, can also facilitate nociceptor-driven immune signalling. These molecules are known to upregulate cytokines, including CCL2 [77] and colony-stimulating factor 1 [78], following neural injury. Thus, nociceptors are integral to modulating pulmonary immunity and influencing the outcomes of bacterial lung infections. Thus, the modulation of neuroimmune crosstalk (e.g. through CGRP antagonism) presents a viable and testable strategy to augment host defences in pneumonia; however, its therapeutic efficacy remains a hypothesis requiring future clinical investigation.

Notably, phenotypic differences in lung clearance and barrier function during pulmonary infection are mediated by distinct neuronal subsets. Recent research has demonstrated that TRPV1 expression in adult DRG is predominantly associated with CGRP+ and SP+ C-fibres [79]. In contrast, Voltage-gated sodium channel Na(v)1.8 is expressed in both myelinated A-fibres and unmyelinated C-fibres [80]. A separate study demonstrated that even when Nav1.8+ sensory neurons were ablated in murine models using diphtheria toxin, CGRP+ neurons expressing TRPV1 were still present [81]. Consequently, future research utilizing more advanced genetic methodologies should aim to delineate the specific functional roles of distinct TRPV1+ and Nav1.8+ neuronal subpopulations in pulmonary immunity and barrier function.

### 5.2. Asthma

**Figure 3.**
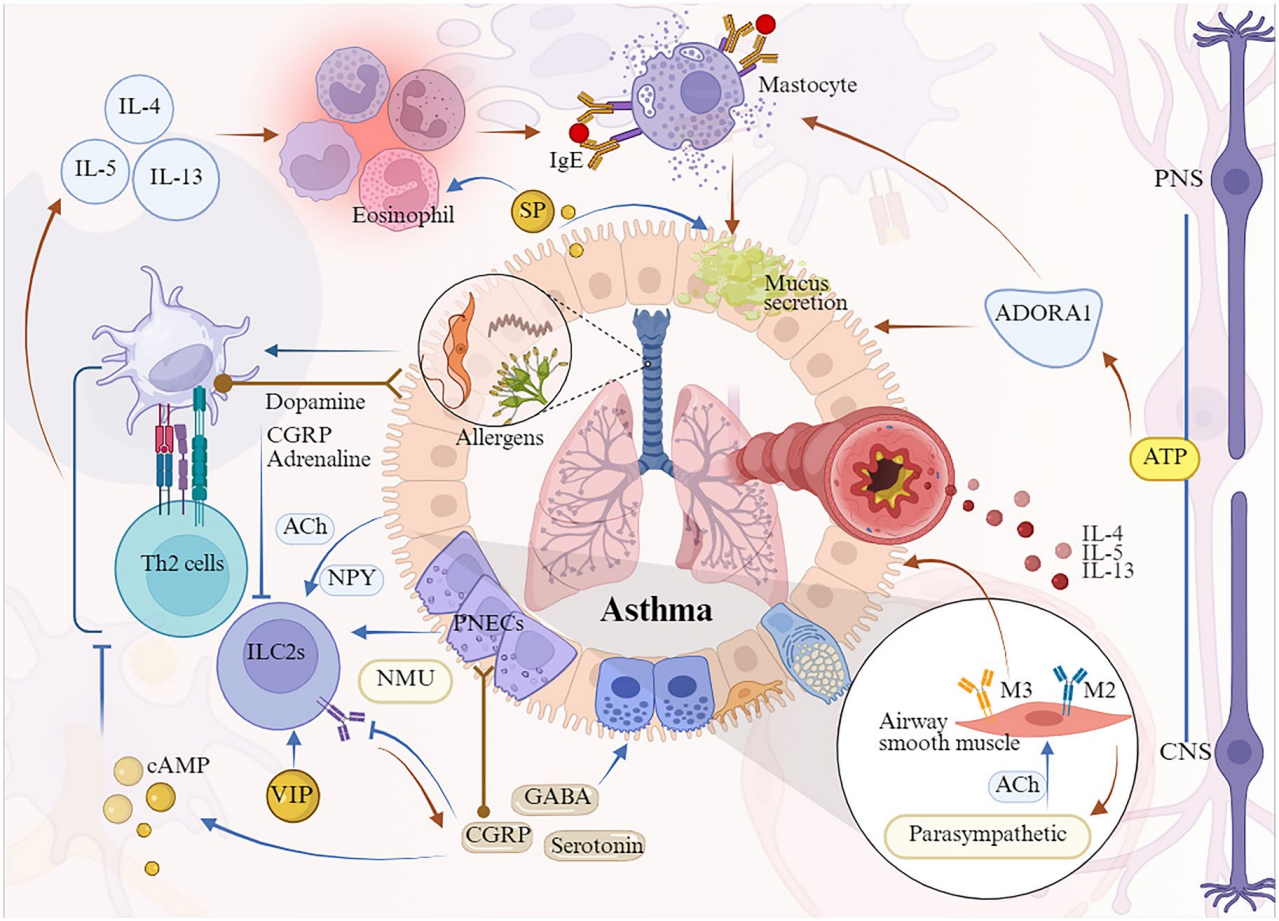
Neuroimmune circuits in asthma pathogenesis. When allergens enter the airways, pulmonary autonomic nerves, and PNECs respond by releasing various neurotransmitters and neuropeptides, including ACh, NMU, VIP, GABA and NPY, which bind to receptors expressed on immune cells. This process recruits Th2 cells, ILC2 cells, macrophages, and eosinophils, triggering IgE class switching, mucous production, and airway hyperresponsiveness, thereby exacerbating asthma symptoms. In contrast, dopamine, norepinephrine, and CGRP may inhibit ILC2-mediated allergic inflammation. Additionally, chronic allergen exposure induces structural and functional remodelling of cholinergic neurons, which is associated with immune cells and inflammatory mediators involved in this process. *Abbreviations:* PNECs, pulmonary neuroendocrine cells; GABA, γ-aminobutyric acid; CGRP, calcitonin gene-related peptide; NMU, neuromedin U; VIP, vasoactive intestinal peptide; cAMP, cyclic AMP; ILC2s, group 2 innate lymphoid cells; ACh, acetylcholine; NPY, neuropeptide Y; Th2 cells, T helper type 2 cells; SP, substance P; PNS, peripheral nervous system; CNS, central nervous system; ADORA1, Adenosine A1 Receptor; ATP, adenosine triphosphate. (Created in BioRender (2025). https://BioRender.com/a46d128).

Bronchial asthma is among the most prevalent chronic respiratory diseases, characterized by symptoms that vary in duration and intensity, including wheezing, shortness of breath, chest tightness and coughing [82]. The disease is marked by bronchial hyperresponsiveness, excessive mucus production, airway narrowing and airway wall remodelling [83]. The initial phase of the allergic response typically involves the uptake of allergens by antigen-presenting cells, leading to the secretion of cytokines and chemokines by airway epithelial cells, which facilitates the recruitment and activation of ILC2s and T helper type 2 CD4+ cells. This immune response initiates the release of type 2 cytokines, such as IL-4, IL-5, and IL-13 [84], which facilitate tissue inflammation by recruiting effector immune cells, including basophils, mast cells and eosinophils, inducing immunoglobulin E (IgE) class switching, as well as promoting mucus production and AHR. These mechanisms are crucial in the onset and intensification of allergic inflammation in asthma. Upon subsequent exposure to allergens, IgE-mediated mast cell degranulation occurs, resulting in the release of histamine and other pro-inflammatory mediators that further aggravate allergic inflammation. Elevated IgE levels and eosinophilia are prominent characteristics of the disease [85]. In recent years, extensive research has considerably enhanced our understanding of neuroimmune interactions in the pathophysiology of asthma. The interaction between the nervous system and immune cells has been shown to play a pivotal role in disease pathogenesis [86] (Figure 3).

In the lungs of asthma patients, there is an increased presence of PNECs, particularly in proximity to ILC2s at airway branch points. PNECs are notably enriched with neurotransmitters such as serotonin and GABA, as well as neuropeptides including SP, neurokinin A, VIP, CGRP and orphanin FQ, which are released in response to allergen exposure [16]. These neurotransmitters and neuropeptides can bind to receptors expressed on ILC2s, transmitting signals from PNECs to ILC2s. Collectively, these components constitute a neuroimmune module that detects and reacts to environmental stimuli entering the airways, thereby directly modulating the immune response. Consequently, the PNEC-ILC2 neuroimmune module serves as a pivotal regulatory unit for allergic responses in the lung [87], suggesting that targeting the neuroimmune interactions within the PNEC-ILC2 axis represents a potential therapeutic strategy for asthma management. Similarly, autonomic nerve fibres in the airways release ACh and NPY to regulate ILC2 activity. These neurotransmitters and neuropeptides play distinct roles in the pathophysiology of asthma. For example, NMU [71] and VIP [88] enhance ILC2 function, whereas CGRP [53] and catecholamines (such as adrenaline and dopamine) [88] suppress ILC2 responses in mucosal tissues.

Research indicates that CGRP functions as a central negative regulator of ILC2-mediated allergic inflammation. In addition to pulmonary sensory neurons and neuroendocrine cells, lung-resident ILC2s uniquely express CGRP and its receptor. Upon binding to its receptor, CGRP induces the production of cyclic adenosine monophosphate (cAMP), which subsequently inhibits antigen presentation and phagocytic activity, as well as reduces the production of pro-inflammatory factors [89]. Moreover, CGRP not only upregulates its expression in ILC2s but also enhances the expression of genes that suppress effector lymphocyte responses [41]. This suggests that by negatively regulating ILC2 responses, CGRP mitigates allergen-induced acute airway inflammation, indicating that activation of the CGRP receptor could serve as a potential therapeutic strategy for allergic asthma. In contrast, another study demonstrated that CGRP can interact with resident ILC2s [87], promoting their maturation and inducing the production of cytokines such as IL-5 and IL-13. IL-5 recruits eosinophils, while IL-13 facilitates the migration of activated pulmonary DCs to draining lymph nodes, where they promote the differentiation of naïve T cells into T-helper 2 (Th2) cells, thereby triggering a cascade of immune responses [90]. The duality of CGRP-mediated effects can be understood by considering the spatiotemporal dynamics of the immune response. CGRP derived from PNECs may act as an early alarmin that initiates and amplifies ILC2 activation upon initial allergen encounter. In contrast, persistent CGRP production by activated ILC2s within established inflammation may initiate a negative feedback loop, potentially *via* receptor desensitization or the induction of specific cAMP-dependent signalling, to prevent excessive tissue damage. The experimental context is also critical: CGRP exhibits a prominent pro-inflammatory role in *in vivo* allergen challenge models, whereas it effectively suppresses ILC2-mediated inflammation in *in vitro* assays. Therefore, the functional outcome is determined by the interplay of these distinct cellular sources and signalling dynamics within the tissue microenvironment, thereby providing a critical framework for informing the development of targeted therapeutic strategies. Additionally, SP not only enhances eosinophil chemotaxis in concert with CGRP but also induces mast cell degranulation and chemotaxis *via* the Mas-related G protein-coupled receptor X2. Recent research has established a direct correlation between eosinophils and increased SP-positive nerve density in asthma. In individuals with asthma, there is an upregulation of both SP and neurokinin-1 receptor (NK1) expression, with the binding of SP to NK1 promoting airway smooth muscle contraction and mucus secretion, thereby contributing to airway obstruction. Notably, SP is expressed not only by neurons but also by immune cells, which are believed to upregulate SP and NK1 expression during asthma. The development of selective antagonists targeting the primary sources of SP in asthma patients is essential for clinical applications [14]. Moreover, the NMU/NMU receptor 1 axis rapidly activates ILC2s and is considered a potential early activator of eosinophilic inflammation in asthma [91]. Although no antagonists targeting NMU signalling have been developed to date, current evidence provides a rationale for the hypothesis that NMU is a therapeutically relevant target in allergic inflammation.

In asthma, GABA contributes to chronic goblet cell hyperplasia and mucus hypersecretion [87]. Conversely, GABA facilitates ASM relaxation, which leads to attenuation of symptoms. Given that GABA acts as an inhibitory neurotransmitter in the CNS, it is imperative that therapeutic strategies selectively target the airways. Therefore, it is crucial to evaluate whether the therapeutic benefits of GABA agonists in asthma outweigh their potential adverse effects.

It is well established that parasympathetic neurons in the airways play a critical role in regulating bronchomotor tone. Parasympathetic cholinergic signalling, facilitated by ACh, induces bronchoconstriction and mucus secretion through the activation of muscarinic acetylcholine receptor M3 (M3R) on ASM and epithelial cells. Concurrently, it provides direct negative feedback to the neurons *via* muscarinic acetylcholine receptor M2 (M2R). Research has documented cholinergic neuronal plasticity in the bronchi of patients with asthma [92]. These findings indicate that chronic exposure to allergens prompts cholinergic neurons to undergo increased branching and elongation, thereby altering neuronal structure and function, which subsequently enhances AHR. BDNF and its receptor, tropomyosin receptor kinase B, are considered pivotal in driving neuronal plasticity. These molecules are expressed by various cell types in the lungs [93], including ASM, fibroblasts, epithelial cells, neurons and immune cells, rendering them potential therapeutic targets to mitigate neuronal remodelling and prevent or alleviate asthma-related symptoms. This is especially important in cases where treatments aimed at reducing airway eosinophils, such as IL-12 or IL-5 antibodies, or even standard inhaled corticosteroids, show limited efficacy in alleviating AHR. We hypothesize that targeting the cholinergic neuronal component could constitute a novel therapeutic strategy for asthma.

The sympathetic nervous system creates a pulmonary environment enriched with dopamine. In individuals with asthma, dopamine levels are inversely associated with the number of circulating ILC2s and compromised lung function. Mechanistic investigations [94] further indicate that dopamine inhibits mitochondrial oxidative phosphorylation in ILC2s, thus suppressing their activity and reducing allergic lung inflammation. Conditional deletion of the dopamine receptor D1 in ILC2s, or chemical ablation of pulmonary dopaminergic neurons, results in heightened ILC2 responses and exacerbated airway inflammation. Therapeutically, localized administration of dopamine reduces ILC2-driven allergic airway inflammation in both *in vitro* and *in vivo* models. These findings elucidate a dopaminergic immune circuit that limits allergic inflammation, presenting a potential therapeutic target for asthma. Recent studies [95] suggest that dopamine, *via* the dopamine receptor D4 in young mice, enhances Th2 cell responses, indicating that the dopamine-dopamine receptor D4 axis may serve as a potential therapeutic target for modulating the progression of allergic asthma from childhood to adulthood. Nonetheless, the comprehensive elucidation of the underlying crosstalk pathways remains incomplete.

Furthermore, an expanding body of evidence indicates that neurons within the peripheral nervous system – including sympathetic, parasympathetic and motor neurons – as well as those in the CNS, employ adenosine triphosphate (ATP) as a neurotransmitter [96]. Extracellular ATP acts as a signalling messenger by activating purinergic receptors, a mechanism referred to as ‘purinergic signalling’, which plays a role in various pathophysiological processes within the lungs and airways [97]. Notably, purinergic receptors are abundantly expressed in the lung and airway tissues, especially within immune cells. As a result, ATP can activate a range of immune cell types, such as eosinophils [98], mast cells [99], DCs [100], and alveolar macrophages [101], thereby directly contributing to airway inflammation. Both ATP and its degradation product, adenosine, have been demonstrated to exert pro-asthmatic effects by stimulating mucin production in epithelial cells [102]. Moreover, adenosine enhances fibronectin expression, which may contribute to airway remodelling. Furthermore, the adenosine receptor A1 has been associated with asthma susceptibility through genome-wide association studies and functional analyses [103], underscoring the potential of purinergic receptors as therapeutic targets for asthma. Future research should aim to elucidate the expression profiles of purinergic receptors in lung and airway tissues to identify more effective therapeutic targets for respiratory diseases. Additionally, further exploration of the regulatory mechanisms underlying purinergic signalling may facilitate the development of novel, targeted treatment strategies for asthma.

### 5.3. COPD

**Figure 4.**
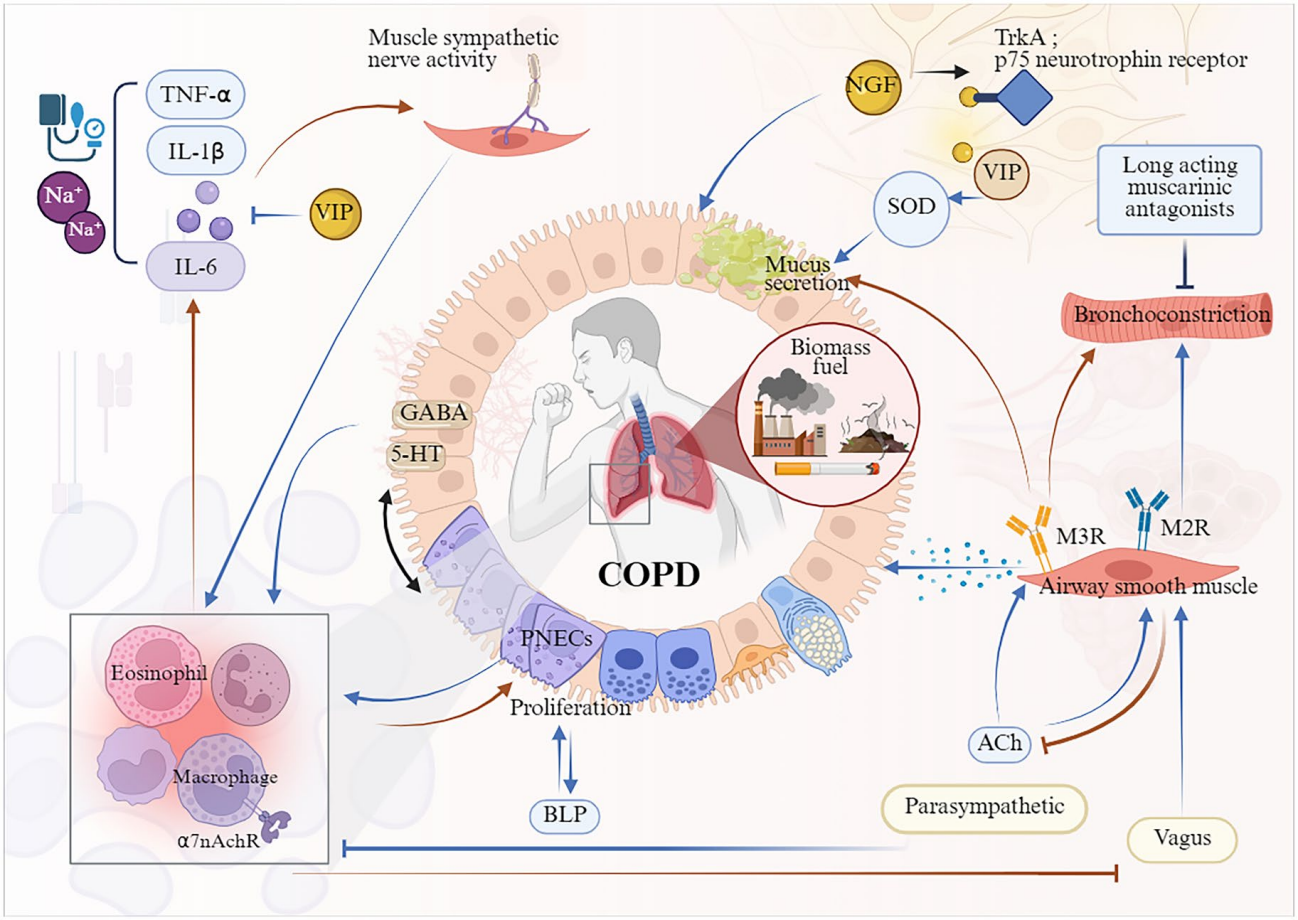
Neuroimmune crosstalk roadmap in COPD. The infiltration of various immune cells constitutes the chronic inflammatory background of COPD, and the inflammatory process is accompanied by increased neural activity. For example, inflammation can promote the release of ACh from pulmonary parasympathetic neurons, utilizing the selective inhibitory effects of ACh in immune regulation to alleviate pulmonary inflammation and damage. VIP can limit the levels of pro-inflammatory cytokines (TNF-α, IL-1β and IL-6) in lung tissue, while increasing SOD, thereby alleviating COPD symptoms through anti-inflammatory and antioxidant mechanisms. In addition, PNECs are involved in regulating the secretion of certain neuropeptides and neurotransmitters, which may trigger abnormal airway remodelling and influence disease progression. *Abbreviations:* PNECs, pulmonary neuroendocrine cells; GABA, γ-aminobutyric acid; BLP, bombesin-like peptide; VIP, vasoactive intestinal peptide; IL-1β, interleukin-1β; IL-6, interleukin-10; TNF-α, tumour necrosis factor-alpha; MSNA, muscle sympathetic nerve activity; NGF, nerve growth factor; TrkA, tropomyosin receptor kinase A; p75NTR, p75 neurotrophin receptor; SOD, superoxide dismutase. (Created in BioRender (2025). https://BioRender.com/e05u282).

COPD is characterized by chronic bronchitis, persistent airway obstruction, airway remodelling and emphysema, leading to a progressive and irreversible decline in lung function. The primary risk factors include exposure to harmful particles and gases, particularly from smoking and biomass fuel inhalation [104]. Similar to asthma, COPD exhibits significant heterogeneity in its inflammatory phenotypes. Chronic inflammation in COPD is marked by the infiltration of inflammatory cells, including neutrophils, macrophages/monocytes, lymphocytes and eosinophils, into the airways and lung tissues. These infiltrations lead to the overexpression of circulating pro-inflammatory markers, including IL-1β, IL-6, TNF-α and C-reactive protein, as well as an increase in total leukocyte counts. This systemic inflammation not only exacerbates the disease but may also impair inspiratory muscle function [105]. Remarkably, elevated muscle sympathetic nerve activity is a characteristic feature of COPD, with inflammation potentially serving as a catalyst for this increased sympathetic activity [106]. This process is associated with elevated circulating NA, which contributes to increased blood pressure and cardiovascular dysfunction. Moreover, the elevation in muscle sympathetic nerve activity has been associated with increased circulating IL-6 levels, suggesting that heightened sympathetic nerve activity in COPD may also contribute to systemic inflammation [106]. These observations suggest a potential bidirectional interaction between inflammatory responses from immune cells and sympathetic nerve activity that regulates respiratory muscles during the progression of COPD (Figure 4). However, the precise mechanisms underlying this neuroimmune crosstalk remain inadequately understood.

The parasympathetic nervous system engages with immune cells through the neurotransmitter ACh, which acts on muscarinic receptors to induce and modulate airway inflammation. Activation of these receptors promotes the release of various cytokines and growth factors that are implicated in the pathology of COPD. Recent research has indicated a reduction in parasympathetic activity among COPD patients, potentially contributing significantly to disease progression [107]. Under chronic inflammatory conditions, pulmonary parasympathetic neurons may become activated, leading to the release of ACh, which interacts with both muscarinic and nicotinic receptors. These receptor subtypes are expressed in lung epithelial and immune cells, including macrophages. ACh exerts selective immunosuppressive effects by regulating immune responses. Elevated ACh levels can activate the α7nAChR on infiltrating inflammatory cells, such as macrophages and neutrophils, thereby inhibiting nuclear factor kappa-light-chain-enhancer of activated B cells (NF-κB) activation and suppressing the secretion of pro-inflammatory cytokines and chemokines by these cells, including alveolar macrophages. This process mitigates lung inflammation and tissue damage [17]. Conversely, vagotomy and α7nAChR deficiency have been shown to exacerbate acute lung injury [108]. Research shows that ACh activates M3R, leading to bronchoconstriction, while simultaneously stimulating inhibitory M2R. This dual action reduces ACh release, establishing a self-regulating negative feedback mechanism that mitigates excessive bronchoconstriction. Conversely, airway inflammation and damage expose vagal nerve terminals to the airway lumen, resulting in the activation of M1R and M3R. This activation promotes bronchoconstriction, mucus secretion, and the initiation of innate inflammatory responses [109]. Recent studies suggest that long-acting muscarinic antagonists, such as tiotropium bromide, may alleviate COPD symptoms by inhibiting airway contraction and providing significant anti-inflammatory effects [110].

In addition, compared to healthy individuals, PNECs are more prevalent in the airways of COPD patients [111]. The increased presence of PNECs may enhance responses to inhaled environmental chemicals, thereby exacerbating airway inflammation and worsening symptoms [112]. PNECs also play a crucial role in modulating immune responses and neuroimmune interactions [113]. Studies have shown that PNECs in COPD may modify the secretion of neuroendocrine factors, such as serotonin, and neuropeptides, such as CGRP, thereby regulating PNEC-dependent alterations in lung chemosensitivity and influencing the progression of the disease [114]. Although the extent to which PNEC hyperactivation exacerbates symptoms in COPD patients remains uncertain, several researchers have hypothesized and empirically demonstrated that bombesin-like peptides secreted by PNECs stimulate the proliferation of human bronchial epithelial cells and lung fibroblasts, resulting in airway thickening and peribronchiolar fibrosis. This pathological airway remodelling is believed to contribute to the progression of COPD [115,116]. Therefore, targeting PNECs is hypothesized to be a viable avenue for the development of new COPD treatments.

Furthermore, levels of the neurotrophic factor NGF and the neuropeptide VIP are elevated in COPD. Stabile et al. demonstrated a correlation between the severity of COPD and increased serum concentrations of NGF. Specifically, serum NGF levels in COPD patients increase in a stage-dependent manner, with a threefold elevation observed in mild to moderate stages and a sixfold elevation in severe to very severe stages [117]. Both *in vivo* and *in vitro* studies indicate that NGF activates downstream effectors, including the tropomyosin receptor kinase A (TrkA), thereby promoting inflammation and tissue remodelling [118]. The bioactivity of NGF is regulated by TrkA and the p75 neurotrophin receptor, both of which are consistently implicated in the progressive inflammatory processes of COPD and may represent potential therapeutic targets. VIP, a neuropeptide extensively expressed in both the nervous and immune systems, plays dual roles in COPD. Serum levels of VIP are elevated during acute exacerbations of COPD [119]; however, recent studies indicate that inhaled VIP therapy may enhance the quality of life for COPD patients [120]. In a mouse model of cigarette smoke-induced COPD, VIP treatment was found to modulate pro-inflammatory cytokines, including tumour TNF-α, IL-1β and IL-6, in lung tissue. Additionally, VIP treatment increased the activity of superoxide dismutase, thereby alleviating symptoms through its anti-inflammatory and antioxidant properties [121]. While the association between elevated serum levels of VIP and the exacerbation of clinical symptoms during acute episodes of COPD remains ambiguous, it is postulated that the increase in VIP may serve as a compensatory response to inflammation and oxidative stress rather than being a direct causative factor of exacerbations. VIP and its derivatives exhibit potential as therapeutic agents for COPD; however, further investigation is required to validate their clinical efficacy and safety. Future research should aim to elucidate the specific regulatory mechanisms of VIP throughout the progression of COPD, thereby enhancing the stability and specificity of VIP-based therapies to optimize their application across various stages of the disease.

### 5.4. Pulmonary fibrosis

**Figure 5.**
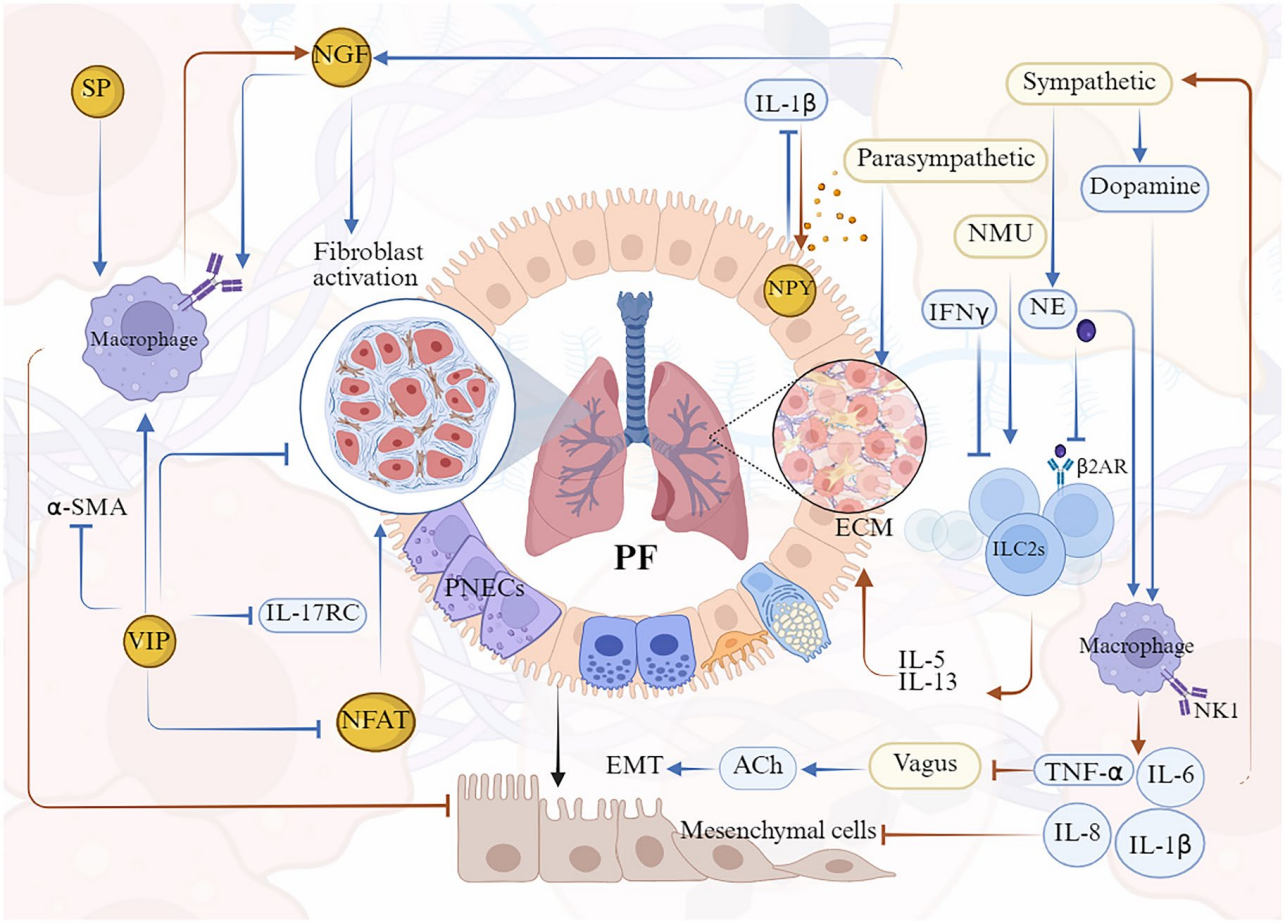
Neuroimmune pathways in PF. Excessive activation of pulmonary myofibroblasts and abnormal ECM deposition are key characteristics of PF. Pulmonary autonomic nerves release various neurotransmitters and neuropeptides, including NE, dopamine, NMU, VIP, NPY and SP, which engage in crosstalk with immune cells (e.g. ILC2s and macrophages) to jointly regulate the progression of PF. For instance, dopamine and NE released by sympathetic nerves, along with inflammatory mediators such as TNF-α, IL-1β, IL-6, and IL-8 secreted by pulmonary macrophages, form a feedback loop. These inflammatory mediators can act on sympathetic nerves to enhance the synthesis and secretion of neurotransmitters, collectively exacerbating pulmonary inflammation and fibrosis. *Abbreviations:* PNECs, pulmonary neuroendocrine cells; EMT, Epithelial-Mesenchymal Transition; NFAT, nuclear factor of activated T cells; VIP, vasoactive intestinal peptide; α-SMA, α-smooth muscle actin; IL-17RC, interleukin-17 Receptor C; ILC2s, type 2 innate lymphoid cells; SP, substance P; NGF, nerve growth factor; IL-1β, interleukin-1β; NPY, neuropeptide Y; NMU, neuromedin U; NE, norepinephrine; β2AR, β2-adrenergic receptor; ECM, extracellular matrix; ACh, acetylcholine; NK1, neurokinin receptor 1; TNF-α, tumour necrosis factor-alpha. (Created in BioRender (2025). https://BioRender.com/l18v511).

Pulmonary fibrosis (PF) is a progressive and irreversible interstitial lung disease with multiple aetiologies. It is characterized by excessive and aberrant deposition of the extracellular matrix (ECM), hyperactivation of fibroblasts and dysregulation of immune cell composition and inflammatory responses [122]. These processes lead to abnormal remodelling of lung architecture and vasculature, impaired gas exchange and ultimately result in respiratory failure and death. IPF represents the most prevalent form of PF [123]. Currently, nintedanib and pirfenidone are the principal antifibrotic agents approved for IPF management. While these medications can decelerate disease progression, they are unable to reverse established fibrosis or cure IPF [124]. Thus, the development of more efficacious therapeutic strategies remains a primary focus of ongoing research. The pathological mechanisms underlying PF involve the release of various mediators, with accumulating evidence indicating that neuroimmune interactions play a crucial role in PF pathogenesis (Figure 5).

Pulmonary myofibroblasts are pivotal effector cells in PF pathogenesis. These cells are not only integral to tissue repair and fibrotic processes but also significantly contribute to the excessive deposition and accumulation of ECM proteins, such as collagen and fibronectin, thereby driving fibrotic tissue formation [125]. The activation and proliferation of pulmonary myofibroblasts are critical factors in lung tissue stiffening and functional decline, with their activity being a decisive factor in determining whether excessive tissue repair progresses to PF [126]. Current antifibrotic agents for PF, such as nintedanib and pirfenidone, achieve their therapeutic efficacy by targeting the activation and differentiation of pulmonary myofibroblasts.

The regulation of alveolar myofibroblasts is mediated by the pulmonary autonomic nervous system and modulated by cytokines derived from immune cells during alveolar formation and differentiation. The autonomic nervous system facilitates the contraction and proliferation of alveolar myofibroblasts through the release of various neurotransmitters [127]. Meanwhile, type 2 cytokines, including TGF-β1, IL-13, and IL-5, secreted by immune cells, stimulate ECM protein production and promote myofibroblast differentiation [128]. Collectively, these factors contribute to the progression of PF. In a murine model of PF deficient in a critical inhibitory factor for ILC2s, the absence of IFN-γ signalling resulted in excessive ILC2 activation during the inflammatory phase, thereby accelerating collagen production by fibroblasts and exacerbating PF [129]. Specifically, ILC2s are significant sources of type 2 cytokines that drive inflammation. Recent studies have elucidated that neuroimmune interactions play a critical role in regulating ILC2 function within mucosal tissues. Evidence establishes that catecholaminergic neurons, which secrete NE and other β2AR ligands, function as modulators that inhibit ILC2 responses. β2AR deficiency exacerbates type 2 lung inflammation and amplifies ILC2 responses, whereas β2AR agonists mitigate inflammation and suppress ILC2 activation [130]. Additionally, the neuropeptide NMU, produced by cholinergic neurons, can activate ILC2s, rapidly inducing the expression of type 2 cytokines (IL-5 and IL-13), thereby exacerbating PF [131,132]. Moreover, alveolar myofibroblasts influence the development and expansion of the pulmonary autonomic nervous system by releasing neurotrophic factors. For example, NGF promotes the activation and migration of pulmonary immune cells, thereby intensifying local inflammation and contributing to PF. In turn, immune cells further stimulate NGF production, creating a positive feedback loop that accelerates the progression of PF [133,134]. This suggests that the nervous system regulates ILC2 responses through multiple pathways, initiating either protective immune responses or promoting inflammatory damage. Meanwhile, the continuous activation of immune cells and cytokine release reciprocally modulate nervous system activity. This intricate neuroimmune crosstalk provides new insights into the pathological mechanisms underlying PF, lending support to the hypothesis that targeting the neuroimmune axis could inhibit fibroblast-to-myofibroblast transformation.

During the pathological progression of PF, the interaction between neuropeptides, neurotransmitters and immune cells plays an important regulatory role. Szema et al. emphasized the critical role of the interaction between VIP and nuclear factor of activated T cells (NFAT) in modulating PF. VIP inhibits NFAT activity, thereby decreasing the release of inflammatory mediators and suppressing immune cell activation. However, in PF patients, VIP levels are frequently reduced, leading to increased NFAT activity, which aggravates lung inflammation and fibrosis [135]. Research conducted by Duan JX’s team demonstrated that VIP could potentially alleviate bleomycin-induced PF in murine models. VIP overexpression resulted in reduced lung tissue damage, ECM deposition and α-smooth muscle actin expression. VIP mitigates epithelial-mesenchymal transition (EMT) by restoring autophagy in alveolar epithelial cells [136] and inhibits fibroblast activation through the downregulation of interleukin-17 receptor C expression [137], thereby effectively attenuating bleomycin-induced PF.

NPY, a polypeptide comprising amino acid residues distributed throughout the nervous system, can interact with various immune cells within the respiratory system and induce EMT in human alveolar epithelial cells. In patients with IPF, NPY is predominantly expressed around bronchial epithelial cells, where it exerts a protective effect against pulmonary fibrosis (PF) by inhibiting the release of IL-1β. Antagonists targeting the Y1 receptor have been shown to suppress IL-1β release while promoting EMT in human alveolar epithelial cells [138]. Therefore, the administration of exogenous NPY or the modulation of its Y1 receptor axis is rationally hypothesized as a potential strategy to slow PF progression, thus warranting further investigation to evaluate its therapeutic efficacy.

SP binds to NK1 receptors on alveolar macrophages, leading to their activation and the subsequent release of inflammatory mediators such as TNF-α and IL-1β. These mediators recruit additional immune cells to the site of inflammation, sustaining and amplifying the inflammatory response. In turn, macrophage-derived inflammatory mediators stimulate nerve terminals, resulting in increased SP release and the establishment of a positive feedback loop that exacerbates lung inflammation and fibrosis [139,140]. Similarly, sympathetic neuron-derived dopamine [141] and NE [142] interact with specific receptors on pulmonary macrophages, thereby stimulating the release of pro-inflammatory mediators such as TNF-α, IL-1β, IL-6, and IL-8. These mediators subsequently enhance neurotransmitter synthesis and secretion within the sympathetic nervous system. This localized neuroimmune interaction establishes a feedforward loop that exacerbates pulmonary inflammation and promotes fibrotic progression. Moreover, macrophage-derived inflammatory mediators can activate the sympathetic nervous system and influence parasympathetic activity. For example, TNF-α inhibits vagal activity, reducing the release of ACh and anti-inflammatory mediators, thereby further exacerbating PF [143,144]. We hypothesize that strategies such as upregulating beneficial neuropeptides, inhibiting the release or receptor binding of harmful neurotransmitters, or targeting relevant signalling pathways could provide promising therapeutic approaches for PF. Nonetheless, further basic research and clinical trials are essential to precisely regulate key nodes within neuroimmune interactions for effective intervention. A comprehensive understanding of the intricate interactions within the lung neuroimmune axis is essential for the development of innovative therapeutic strategies.

### 5.5. Lung cancer

**Figure 6.**
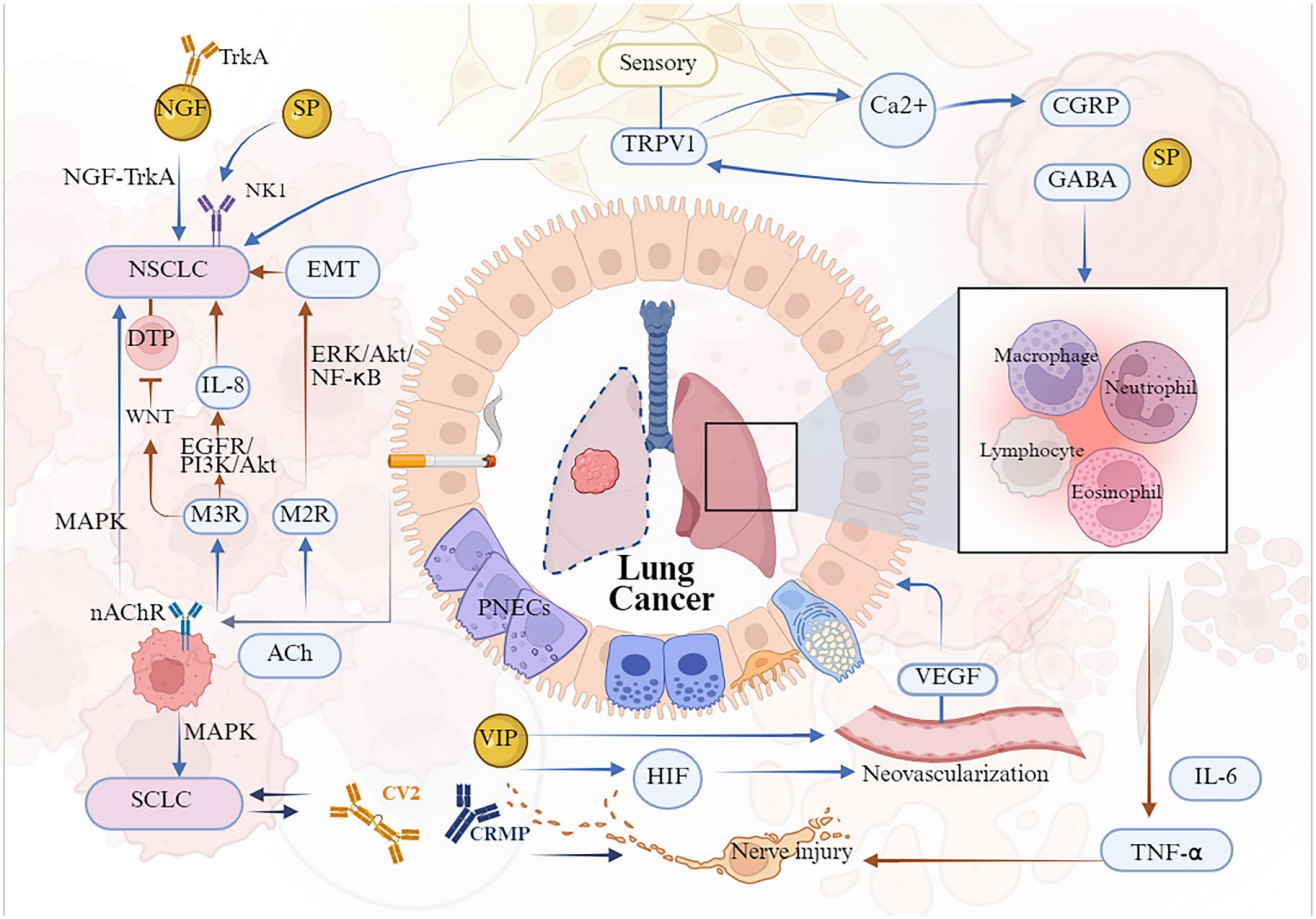
Neuroimmune networks in lung cancer. As components of the tumour microenvironment, the complex interactions between the nervous and immune systems influence the initiation and progression of lung cancer. The release of neurotransmitters regulates the recruitment and differentiation of immune cells, promotes cytokine secretion, and affects the proliferation, migration and invasion of lung cancer cells. For example, ACh activates the EGFR/PI3K/AKT and ERK/Akt/NF-κB signalling pathways through M3R and M2R, respectively, upregulating the secretion of the pro-inflammatory cytokine IL-8 and inducing EMT, thereby accelerating the progression of NSCLC. Additionally, ACh induces the proliferation of SCLC cells via pathways such as MAPK, and promotes the production of various autoantibodies, including anti-CRMP and anti-CV2 antibodies, which erroneously attack normal neurons. Moreover, cytokines released by the activated immune system, such as TNF-α and IL-6, further exacerbate the inflammatory response and neural damage, contributing to the deterioration and complications of lung cancer. *Abbreviations:* PNECs, pulmonary neuroendocrine cells; VIP, vasoactive intestinal peptide; HIF, hypoxia-inducible factor; VEGF, vascular endothelial growth factor; SCLC, small cell lung cancer; ACh, acetylcholine; NSCLC, non-small cell lung cancer; nAChRs, nicotinic acetylcholine receptors; M2R, M2 muscarinic receptor; M3R, M3 muscarinic receptor; EMT, Epithelial-Mesenchymal Transition; NK1, neurokinin receptor 1; GABA, γ-aminobutyric acid; CGRP, calcitonin gene-related peptide; SP, substance P; TNF-α, tumour necrosis factor-alpha. (Created in BioRender (2025). https://BioRender.com/a05j435).

Lung cancer, recognized as one of the most prevalent malignancies, is characterized by complex and not fully elucidated biological processes that facilitate uncontrolled cellular proliferation and metastasis. Consequently, it remains a leading cause of cancer-related mortality, responsible for approximately 18% of all cancer deaths [145]. Lung cancer is generally categorized into two primary histological subtypes: small cell lung cancer (SCLC), which constitutes approximately 15% of all primary lung cancer cases, and non-small cell lung cancer (NSCLC), which includes various forms such as squamous cell carcinoma and adenocarcinoma, representing 80-85% of cases [146,147]. Increasing research efforts are directed towards elucidating the underlying mechanisms of lung cancer pathogenesis, identifying molecular targets on cell surfaces, and devising therapeutic interventions. As a component of the tumour microenvironment, the nervous system plays a pivotal role in tumour initiation and progression, engaging in complex interactions with the immune system. In the context of lung cancer research, the interaction between nerves and tumour cells has shown potential for modulating tumour progression and mitigating complications through specific pathways (Figure 6).

Neurotransmitters are increasingly acknowledged as vital elements of the tumour microenvironment, influencing a range of malignancy-associated phenotypes [148]. The TRPV1 is primarily expressed in afferent sensory neurons and is also extensively present in various tumour cells [149]. Activation of TRPV1 has been shown to induce an increase in intracellular Ca^2+^ influx, which subsequently activates downstream Ca^2+^ signalling pathways [150], mediating the release of neurotransmitters such as CGRP, SP, GABA and somatostatin [151,152]. These neurotransmitters can modulate the recruitment, differentiation and cytokine production of immune cells within the tumour microenvironment [153]. For example, Wang et al. demonstrated that TRPV1 expression is significantly upregulated in NSCLC, and TRPV1 knockout markedly suppressed tumour growth in immunocompetent mice. This suppression was attributed to a reduction in GABA secretion in NSCLC, which decreased immune cell infiltration and enhanced the antitumor immune response [154]. Moreover, B cells are capable of synthesizing and secreting GABA, thereby contributing to the regulation of antitumor immune responses [155].

Epidemiological evidence demonstrates a significant aetiological link between smoking and the onset of all histological types of lung cancer [156]. Nicotine, a highly addictive constituent of cigarettes, facilitates tumour cell growth through nicotinic acetylcholine receptors (nAChR) [157]. Studies have shown that the release of ACh activates the MAPK pathway, the intracellular calcium pathway, and the Akt phosphorylation pathway in human SCLC cells [158], thereby promoting lung cancer cell growth and proliferation. Similarly, ACh is closely associated with the upregulation of NSCLC cell proliferation, angiogenesis, migration and invasion. Lin et al. [159] demonstrated that ACh promotes NSCLC cell proliferation through M3R-mediated activation of the MAPK pathway. Additionally, M3R upregulates the transcription of matrix metalloproteinases and EMT-related genes *via* the PI3K/Akt pathway, resulting in decreased E-cadherin levels and enhancing adhesion and migration in NSCLC cell lines. Xu et al. [160] found that ACh activates the epidermal growth factor receptor (EGFR)/PI3K/Akt signalling pathway in a dose-dependent manner *via* the M3R, leading to the upregulation of pro-inflammatory factor IL-8, thereby promoting NSCLC cell proliferation, invasion and migration. Therefore, we propose a testable therapeutic hypothesis that downregulating M3R, inhibiting the EGFR/PI3K/Akt pathway, and blocking IL-8 signalling constitutes a rational multi-target strategy to disrupt the NSCLC tumour microenvironment. Zhao et al. [161] identified the M2R/ERK/Akt/NF-κB axis as a potential therapeutic target for NSCLC. Inhibition of M2R, either through antagonists or siRNA, effectively reduces the activity of the PI3K/Akt, MAPK, ERK and NF-κB p65 pathways, thereby impeding NSCLC cell migration, invasion and EMT. It is well-established that EGFR-tyrosine kinase inhibitor (TKI) therapy is an efficacious treatment for EGFR-mutant NSCLC [162]. However, drug-tolerant persister (DTP) cells, which survive initial treatment, eventually contribute to therapeutic resistance, with ACh signalling playing a crucial role in this process. Nie et al. [163] revealed that EGFR-TKI therapy upregulates the synthesis and secretion of ACh in DTP cells. This process activates the downstream muscarinic signalling pathway, facilitating the nuclear translocation of WNT ligands and β-catenin, which ultimately leads to the activation of the WNT pathway and the development of drug tolerance. Targeting the ACh/M3R/WNT axis represents a rational therapeutic hypothesis for patients undergoing EGFR-TKI treatment, with the potential to overcome DTP cell formation, enhance EGFR-TKI efficacy and reduce tumour recurrence. Notably, non-tumour cells within the tumour microenvironment, including immune cells such as T cells, B cells and macrophages, are also known to synthesize and release ACh, thereby exerting immunomodulatory effects [164,165].

As critical signalling molecules, neuropeptides not only participate in the proliferation and migration of lung cancer cells but also influence immune cell behaviour, angiogenesis and neural components within the tumour microenvironment [166]. Casibang et al. [167] reported that VIP receptors are present in 58% of lung cancer biopsy samples. VIP promotes lung cancer cell proliferation through the secretion of bombesin-like peptides and neurotensin, as well as by upregulating the expression of vascular endothelial growth factor mRNA. The continuous proliferation of cancer cells results in high oxygen consumption and VIP can activate hypoxia-inducible factor (HIF). The continuous proliferation of cancer cells consumes large amounts of oxygen and VIP can activate HIF [168], thereby promoting angiogenesis and tumour metastasis [169]. Evidence suggests that following VIP treatment, HIF levels in lung adenocarcinoma cells return to baseline, which may have therapeutic implications for cancer treatment [170]. Additionally, NGF is known to be expressed in tumour cells, promoting neural regeneration and differentiation within the tumour microenvironment [171]. These nerve fibres not only furnish nutritional support to the tumour but also secrete neurotransmitters, such as NE, which enhance tumour cell proliferation and angiogenesis [172]. The α1-adrenergic receptor (α1AR) is expressed in immune cells and regulates their activation within the tumour microenvironment, affecting both their migration and the production of inflammatory cytokines. The release of cytokines, such as TNF-α and IL-6, subsequently augments NGF production [173], and this reciprocal interaction collectively fosters tumour growth and metastasis. Moreover, research has indicated that NGF and its receptor TrkA are upregulated in NSCLC and targeting the NGF-TrkA axis with specific lung cancer therapies may alleviate symptoms of squamous cell carcinoma [174]. SP is a neuropeptide secreted by both the nervous and immune systems, recognized for its role in pain perception and its contribution to the induction and maintenance of chronic pain in cancer patients. Yang et al. [175] demonstrated that SP upregulates TLR4 expression by interacting with the NK1 receptor on tumour cells, thereby promoting tumour cell proliferation, migration and invasion. Previous studies have reported that silencing TLR4 suppresses lung cancer metastasis [176]. Concurrently, TLR4 expression has been correlated with clinical staging, pathological grading and chemotherapy resistance in malignant tumours [177,178]. Therefore, we hypothesize that targeting the SP/NK1 pathway to reduce TLR4 expression could serve as an effective adjunctive therapy for lung cancer. The findings imply that pharmacological interventions aimed at these neurotrophic and immune signalling pathways could potentially inhibit neurogenesis and its stimulatory effects on cancer progression and pain. This hypothesis merits rigorous clinical validation to evaluate its efficacy in mitigating disease progression and alleviating cancer-associated pain.

Additionally, SCLC, the most aggressive subtype of lung cancer, is closely associated with several paraneoplastic neurological autoimmune disorders, such as autoimmune encephalomyelitis [179]. Neurons, immune cells and their secreted cytokines contribute to the pathogenesis of these complications. SCLC expresses neuron-specific antigens that trigger the T cell-mediated immune response, resulting in the production of several autoantibodies, including anti-collapsin response mediator protein/anti-CV2 antibodies, anti-Hu antibodies and anti-amphiphysin antibodies [180]. These autoantibodies not only target cancer cells but also mistakenly recognize and attack neurons in normal regions of the CNS, resulting in distal neuronal damage and various pathological changes. Additionally, the release of pro-inflammatory cytokines, such as TNF-α and IL-6, following immune system activation exacerbates neural injury and inflammation [181]. Despite some advancements, effective treatments for SCLC and its associated paraneoplastic neurological syndromes remain a significant challenge. Future research should aim to elucidate the specific roles of neurons and immune cells in SCLC to develop more targeted therapeutic strategies. For instance, the integration of neuroprotective agents with immunomodulatory therapies holds the potential for effectively mitigating the symptoms of paraneoplastic neurological disorders and enhancing patient quality of life.

## 6. Research limitations and translational challenges

Despite the promising prospects, current research is constrained by limitations and potential controversies across various dimensions. It is noteworthy that the majority of findings in pulmonary neuroimmunology are derived from preclinical studies, which primarily focus on the mechanistic exploration of neuroimmune interactions. Additionally, the current literature may be subject to publication bias, wherein positive findings concerning neuroimmune pathways could influence our understanding of their actual role in disease. In contrast, research on clinical translation remains relatively underdeveloped. Reports on selective agonists or antagonists targeting candidate molecules within the pulmonary neuroimmune axis for lung diseases that have advanced to phase III clinical trials are particularly scarce. Relevant animal studies indicate that the CGRP receptor antagonist, olcegepant, significantly suppresses CGRP-overrelease-induced activation of ILC2s, thereby mitigating airway inflammation and the progression of allergic asthma in murine models [15]. Similarly, the neurokinin 1 receptor antagonist aprepitant has been shown to inhibit SP-induced pulmonary inflammation and fibrosis in rat models of pulmonary fibrosis [182]. Additionally, TRPV4, an ion channel expressed on sensory nerves, and its antagonist have been demonstrated to reduce IL-8 levels and inhibit fibroblast activation in rat models of pulmonary fibrosis [183]. Notably, for many other promising targets discussed in this review (e.g. NMU, NPY), there is still a lack of robust *in vivo* validation in human disease models. Despite the promising findings in the field, translating the mechanisms of neuroimmune interactions into effective clinical therapies remains challenging. A major issue is that existing research models, including animal and *in vitro* systems, often fail to accurately replicate the complexity and dynamics of pulmonary neuroimmune interactions as they manifest in human diseases. Additionally, interspecies differences, such as variations in the anatomical structure and innervation patterns of the vagus nerve, which are not entirely consistent between experimental animals and humans, may present a significant obstacle in drug development [184]. Moreover, the high heterogeneity of patients in clinical trials can obscure the identification of beneficial subgroups, and there is a notable lack of reliable biomarkers and novel clinical endpoints that are sensitive to the effects of neuroimmune modulation. For patient populations with diverse genetic backgrounds, sexes and ages, it is questionable whether traditional pulmonary disease endpoints, such as forced expiratory volume in 1 second, forced vital capacity and patient-reported outcomes, are adequate to capture the benefits conferred by neuroimmune modulation. This raises the question of whether new biomarkers need to be defined, such as neuroactivity imaging or specific immune cell or neuropeptide profiles. Answers to these questions remain within the current knowledge gap. In addition, key molecular targets involved in neuroimmune circuits may exhibit functional duality. For instance, CGRP can exert either promotive or inhibitory effects on neurogenic inflammation and immune responses in a concentration-dependent manner [185], thereby complicating precise intervention strategies in drug development. More profoundly, the intricate neuroimmune crosstalk network in humans encompasses numerous interconnected signalling pathways. Targeting a single molecular entity may lead to compensatory regulation by associated pathways, potentially affecting the efficacy of the intervention or causing dysfunction in other aspects of the host’s neural function or immune defence. Research indicates that TRPV1 antagonists can suppress cough and inflammation induced by neuropeptide release from airway sensory nerves, suggesting their potential as therapeutics for allergic cough [186,187]; while they carry the side effect of inducing thermoregulatory dysfunction in humans [188]. Furthermore, the precise delivery of drugs to specific neuroimmune interaction sites within the lungs, while avoiding potential issues of off-target effects or insufficient dosing, represents another critical challenge that urgently requires scientific resolution.

## 7. Conclusions and perspectives

The global burden of prevalent respiratory diseases continues to rise, impacting hundreds of millions of individuals annually and resulting in at least four million premature deaths [189,190]. Despite advancements in therapeutic strategies over the past decades, challenges such as increasing drug resistance, lack of precision and treatment limitations persist as significant barriers within the current therapeutic framework. An expanding body of research is transitioning from an organ-centric viewpoint to a focus on systemic neuroimmune network regulation. Consequently, this review systematically elucidates the critical role of bidirectional neuroimmune interactions in regulating pulmonary homeostasis and influencing disease progression. Specifically, the bidirectional communication between airway neurons – such as the pulmonary branches of the vagus nerve and TRPV1-positive sensory nerves – and resident immune cells – including alveolar macrophages and PNECs – is mediated by diverse neurotransmitters (NE, ACh, dopamine, GABA, serotonin, glutamate), neuropeptides (CGRP, SP, NMU, NPY, VIP, NGF), and immune factors (TNF, ILs). In the context of pulmonary infections, asthma, COPD, PF and lung cancer, the dysregulation of the pulmonary neuroimmune axis underlies shared pathological processes. These processes include the aberrant release of neurotransmitters or neuropeptides, dysregulated immune cell responses, and sensory nerve sensitization. Simultaneously, a cross-disease perspective reveals distinct phenotypic characteristics. For example, the activation of the ‘SP-alveolar macrophage – TNF-α/IL-1β’ circuit exacerbates lung fibrogenesis in pulmonary fibrosis [139,140]. In contrast, in lung cancer, macrophage-derived NGF within the tumour microenvironment promotes tumour cell growth and neoangiogenesis, drives tumour neurogenesis, and induces cancer-related pain [171–173]. The identification of these shared and disease-specific pathways provides a clear roadmap for future research. Targeting key molecules and signalling pathways within the neuroimmune axis presents promising opportunities for discovering diagnostic biomarkers and developing pathogenesis-based precision therapeutic strategies.

Encouragingly, the translational potential of these mechanisms is demonstrated by several neuroimmune-targeting agents that have successfully reached clinical use or trials. For instance, the M3 receptor antagonist tiotropium bromide effectively inhibits airway constriction and inflammation induced by ACh release from airway nerve terminals in COPD patients, with clinical trials indicating its favourable tolerability [191]. Exposure to IL-5 markedly enhances sensory nerve density within the airway epithelium and eosinophils, increasing the risk of airway hyperreactivity [192]. Mepolizumab, a humanized monoclonal antibody targeting IL-5, has been shown to effectively inhibit eosinophilic inflammation, leading to a reduction in exacerbations and a decrease in the need for systemic corticosteroids in patients with severe asthma [193]. Besides, gefapixant, recognized as the first targeted antitussive for refractory or unexplained chronic cough in adults, functions by inhibiting extracellular ATP signalling through the blockade of P2X3 receptors on airway vagal C-fibres, thus mitigating cough and airway inflammation induced by sensory nerve activation [194]. Despite its approval in Japan and the European Union, the FDA declined its New Drug Application, citing ‘lack of substantial evidence of effectiveness’ in phase III trials [195]. Moreover, the potential for dysgeusia as an adverse event presents additional challenges for the broader deployment of this targeted therapy in other regions. The successful application of the IL-5 inhibitor mepolizumab serves as a paradigm for translating neuroimmune crosstalk mechanisms into precision therapies, offering valuable insights for the treatment of respiratory diseases. It is important to highlight that the current paucity of clinical translation data underscores the significant developmental potential within this domain. Emerging research reports that pneumonia activates GABAergic neurons in the brain, leading to the release of NE from pulmonary sympathetic nerves [196]. This process subsequently activates local ADRB2-expressing macrophages in the lungs, exacerbating pulmonary cytokine storms and tissue damage. In contrast, targeted inhibition of this brain-lung neuroimmune axis has been shown to improve clinical outcomes in severe pneumonia, indicating its potential as an innovative therapeutic strategy for this condition [196]. As illustrated in Table 2, our work has identified the mechanistic potential of multiple key targets, necessitating further drug formulation development and clinical trials to advance them to clinical candidate stages.

To overcome existing challenges and translate these mechanistic insights into viable therapies, concerted multidisciplinary collaborations integrating neuroscience, immunology, pulmonology, bioengineering and clinical pharmacology are essential. A key priority is to advance the necessary *in vivo* and clinical studies to validate these promising neuroimmune targets. In this context, several emerging technologies are poised to facilitate breakthroughs in understanding neuroimmune crosstalk, unlock translational pathways, and accelerate the development of potential drugs or novel therapies. To tackle species differences in research models, humanized lung organoid models have shown unique advantages [197]. These models integrate neurons differentiated from human induced pluripotent stem cells, various immune cells, and lung epithelial cells to simulate the pulmonary neuroimmune microenvironment. When combined with microfluidic chip technology [198], these models facilitate more accurate high-throughput drug screening. For *in vivo* mechanistic analysis, the integration of optogenetics with two-photon microscopy may offer real-time visualization tools for observing neuroimmune interactions within disease models [199], thereby providing direct experimental evidence to support the development of new therapies. Concurrently, the application of single-cell multi-omics or spatial transcriptomics to map disease stage-specific and region-specific (such as airway versus alveoli, lesion core versus periphery) neuroimmune interaction profiles in lung diseases [200], in conjunction with advanced technologies like artificial intelligence and algorithmic modelling, will aid in identifying potential biomarkers, constructing predictive models, and enhancing patient stratification and personalized therapy. Additionally, the development of innovative bioadhesive nanoparticles designed to selectively deliver molecular agonists or antagonists to the pulmonary neuroimmune axis presents a promising approach to maintaining local drug concentrations while minimizing systemic side effects [201]. Notably, recent studies have uncovered extensive interactions between the lung microbiome and distal neuroimmune networks mediated by the pulmonary microbiome [202]. This novel insight prompts the question: Could intervention strategies targeting the microbiome to modulate the pulmonary neuroimmune network be significant for the treatment of respiratory diseases? This area may represent a promising avenue for future research in the field.

In conclusion, by decoding the intricate network of pulmonary neuroimmune crosstalk, this review provides novel pathophysiological insights and establishes a foundational framework for future research and therapeutic innovation in respiratory medicine. The application of advanced technologies, including organoid models, single-cell multi-omics and spatial transcriptomics, presents significant potential for translating the understanding of molecular mechanisms governing bidirectional neuroimmune communication into precise therapeutic targets [203]. This advancement paves the way for stratified diagnostic and treatment approaches, as well as personalized intervention strategies, in the management of pulmonary diseases.

## Data Availability

Data sharing is not applicable to this article as no data were created or analyzed in this study.

## References

[1] Karlsson JA, Sant’Ambrogio G, Widdicombe J. Afferent neural pathways in cough and reflex bronchoconstriction. J Appl Physiol (1985). 1988;65(3):1007–1023. doi: 10.1152/jappl.1988.65.3.1007. 3053580

[2] Elenkov IJ, Wilder RL, Chrousos GP, et al. The sympathetic nerve–an integrative interface between two supersystems: the brain and the immune system. Pharmacol Rev. 2000;52(4):595–638. doi: 10.1016/S0031-6997(24)01470-4. 11121511

[3] Chen J, Lai X, Song Y, et al. Neuroimmune recognition and regulation in the respiratory system. Eur Respir Rev. 2024;33(172):240008. doi: 10.1183/16000617.0008-2024. 38925790 PMC11216688

[4] Steinman L. Elaborate interactions between the immune and nervous systems. Nat Immunol. 2004;5(6):575–581. doi: 10.1038/ni1078. 15164017

[5] Kaye AD, Perilloux DM, Hawkins AM, et al. Tumor necrosis factor and interleukin modulators for pathologic pain states: a narrative review. Pain Ther. 2024;13(3):481–493. doi: 10.1007/s40122-024-00603-8. 38724743 PMC11111651

[6] Mueller KL, Hines PJ, Travis J. Neuroimmunology. Science (1979). 2016;353(6301):760–761. doi: 10.1126/science.353.6301.760. 27540161

[7] Biltz RG, Sawicki CM, Sheridan JF, et al. The neuroimmunology of social-stress-induced sensitization. Nat Immunol. 2022;23(11):1527–1535. doi: 10.1038/s41590-022-01321-z. 36369271 PMC10000282

[8] Jassam YN, Izzy S, Whalen M, et al. Neuroimmunology of traumatic brain injury: time for a paradigm shift. Neuron. 2017;95(6):1246–1265. doi: 10.1016/j.neuron.2017.07.010. 28910616 PMC5678753

[9] Caldwell LJ, Subramaniam S, MacKenzie G, et al. Maximizing the potential of neuroimmunology. Brain Behav Immun. 2020;87:189–192. doi: 10.1016/j.bbi.2020.03.010. 32201255 PMC8353661

[10] Warr MR, Pietras EM, Passegué E. Mechanisms controlling hematopoietic stem cell functions during normal hematopoiesis and hematological malignancies. Wiley Interdiscip Rev Syst Biol Med. 2011;3(6):681–701. doi: 10.1002/wsbm.145. 21412991

[11] Méndez-Ferrer S, Michurina TV, Ferraro F, et al. Mesenchymal and haematopoietic stem cells form a unique bone marrow niche. Nature. 2010;466(7308):829–834. doi: 10.1038/nature09262. 20703299 PMC3146551

[12] Gergues M, Nagula V, Bliss SA, et al. Neuroimmune/hematopoietic axis with distinct regulation by the high-mobility group box 1 in association with tachykinin peptides. J Immunol. 2020;204(4):879–891. doi: 10.4049/jimmunol.1900582. 31924647 PMC6994841

[13] Guan WJ, Zheng XY, Chung KF, et al. Impact of air pollution on the burden of chronic respiratory diseases in China: time for urgent action. Lancet. 2016;388(10054):1939–1951. doi: 10.1016/s0140-6736(16)31597-5. 27751401

[14] Azzoni R, Perdijk O, Harris NL, et al. Neuroimmunology of the lung. Annu Rev Immunol. 2024;42(1):57–81. doi: 10.1146/annurev-immunol-083122-042512. 37989144

[15] Xie X, Li Y, Yan B, et al. Mediation of the JNC/ILC2 pathway in DBP-exacerbated allergic asthma: a molecular toxicological study on a neuroimmune positive feedback mechanism. J Hazard Mater. 2024;465:133360. doi: 10.1016/j.jhazmat.2023.133360. 38157815

[16] Pavón-Romero GF, Serrano-Pérez NH, García-Sánchez L, et al. Neuroimmune pathophysiology in asthma. Front Cell Dev Biol. 2021;9:663535. doi: 10.3389/fcell.2021.663535. 34055794 PMC8155297

[17] Pinheiro NM, Santana FP, Almeida RR, et al. Acute lung injury is reduced by the α7nAChR agonist PNU-282987 through changes in the macrophage profile. Faseb J. 2017;31(1):320–332. doi: 10.1096/fj.201600431R. 27729414

[18] Yuan L, Tao J, Wang J, et al. Global, regional, national burden of asthma from 1990 to 2021, with projections of incidence to 2050: a systematic analysis of the global burden of disease study 2021. EClinicalMedicine. 2025;80:103051. doi: 10.1016/j.eclinm.2024.103051. 39867965 PMC11764843

[19] Naeem S, Wang F, Mubarak R, et al. Mapping the global distribution, risk factors, and temporal trends of COPD incidence and mortality (1990–2021): ecological analysis. BMC Med. 2025;23(1):210. doi: 10.1186/s12916-025-04014-0. 40197280 PMC11977891

[20] Song JW, Hong SB, Lim CM, et al. Acute exacerbation of idiopathic pulmonary fibrosis: incidence, risk factors and outcome. Eur Respir J. 2011;37(2):356–363. doi: 10.1183/09031936.00159709. 20595144

[21] Bray F, Laversanne M, Sung H, et al. Global cancer statistics 2022: GLOBOCAN estimates of incidence and mortality worldwide for 36 cancers in 185 countries. CA Cancer J Clin. 2024;74(3):229–263. doi: 10.3322/caac.21834. 38572751

[22] Aven L, Ai X. Mechanisms of respiratory innervation during embryonic development. Organogenesis. 2013;9(3):194–198. doi: 10.4161/org.24842. 23974176 PMC3896590

[23] Belvisi MG. Overview of the innervation of the lung. Curr Opin Pharmacol. 2002;2(3):211–215. doi: 10.1016/s1471-4892(02)00145-5. 12020459

[24] Carr MJ, Undem BJ. Bronchopulmonary afferent nerves. Respirology. 2003;8(3):291–301. doi: 10.1046/j.1440-1843.2003.00473.x. 14528878

[25] Suarez-Mier GB, Buckwalter MS. Glial fibrillary acidic protein-expressing glia in the mouse lung. ASN Neuro. 2015;7(5):1759091415601636. doi: 10.1177/1759091415601636. 26442852 PMC4601129

[26] Seguella L, Gulbransen BD. Enteric glial biology, intercellular signaling and roles in gastrointestinal disease. Nat Rev Gastroenterol Hepatol. 2021;18(8):571–587. doi: 10.1038/s41575-021-00423-7. 33731961 PMC8324524

[27] Tang M, Xu M, Wang J, et al. Brain metastasis from EGFR-mutated non-small cell lung cancer: secretion of IL11 from astrocytes up-regulates PDL1 and promotes immune escape. Adv Sci (Weinh). 2024;11(26):e2306348. doi: 10.1002/advs.202306348. 38696655 PMC11234401

[28] Kiaie N, Gorabi AM, Loveless R, et al. The regenerative potential of glial progenitor cells and reactive astrocytes in CNS injuries. Neurosci Biobehav Rev. 2022;140:104794. doi: 10.1016/j.neubiorev.2022.104794. 35902044

[29] Zhou Y, Zhang Y, Xu J, et al. Schwann cell-derived exosomes promote lung cancer progression via miRNA-21-5p. Glia. 2024;72(4):692–707. doi: 10.1002/glia.24497. 38192185

[30] Shurin MR, Wheeler SE, Shurin GV, et al. Schwann cells in the normal and pathological lung microenvironment. Front Mol Biosci. 2024;11:1365760. doi: 10.3389/fmolb.2024.1365760. 38638689 PMC11024312

[31] Heredia JE, Jung M, Balestrini A, et al. Single-cell transcriptomic analysis links nonmyelinating Schwann cells to proinflammatory response in the lung. J Immunol. 2023;211(5):844–852. doi: 10.4049/jimmunol.2200946. 37477665 PMC10450159

[32] Lloyd CM, Marsland BJ. Lung homeostasis: influence of age, microbes, and the immune system. Immunity. 2017;46(4):549–561. doi: 10.1016/j.immuni.2017.04.005. 28423336

[33] Vivier E, Artis D, Colonna M, et al. Innate lymphoid cells: 10 years on. Cell. 2018;174(5):1054–1066. doi: 10.1016/j.cell.2018.07.017. 30142344

[34] Joffre OP, Segura E, Savina A, et al. Cross-presentation by dendritic cells. Nat Rev Immunol. 2012;12(8):557–569. doi: 10.1038/nri3254. 22790179

[35] Godinho-Silva C, Cardoso F, Veiga-Fernandes H. Neuro-immune cell units: a new paradigm in physiology. Annu Rev Immunol. 2019;37(1):19–46. doi: 10.1146/annurev-immunol-042718-041812. 30379595

[36] Shouman K, Benarroch EE. Peripheral neuroimmune interactions: selected review and some clinical implications. Clin Auton Res. 2021;31(4):477–489. doi: 10.1007/s10286-021-00787-5. 33641054 PMC7914391

[37] Berthoud HR, Neuhuber WL. Functional and chemical anatomy of the afferent vagal system. Auton Neurosci. 2000;85(1-3):1–17. doi: 10.1016/s1566-0702(00)00215-0. 11189015

[38] Morelli AE, Sumpter TL, Rojas-Canales DM, et al. Neurokinin-1 receptor signaling is required for efficient Ca(2+) flux in T-cell-receptor-activated T cells. Cell Rep. 2020;30(10):3448–3465.e8. doi: 10.1016/j.celrep.2020.02.054. 32160549 PMC7169378

[39] Fattori V, Ferraz CR, Rasquel-Oliveira FS, et al. Neuroimmune communication in infection and pain: friends or foes? Immunol Lett. 2021;229:32–43. doi: 10.1016/j.imlet.2020.11.009. 33248166

[40] Holzmann B. Antiinflammatory activities of CGRP modulating innate immune responses in health and disease. Curr Protein Pept Sci. 2013;14(4):268–274. doi: 10.2174/13892037113149990046. 23745695

[41] Wallrapp A, Burkett PR, Riesenfeld SJ, et al. Calcitonin gene-related peptide negatively regulates alarmin-driven type 2 innate lymphoid cell responses. Immunity. 2019;51(4):709–723.e6. doi: 10.1016/j.immuni.2019.09.005. 31604686 PMC7076585

[42] Duan JX, Zhou Y, Zhou AY, et al. Calcitonin gene-related peptide exerts anti-inflammatory property through regulating murine macrophages polarization in vitro. Mol Immunol. 2017;91:105–113. doi: 10.1016/j.molimm.2017.08.020. 28892747

[43] Du J, Li XH, Li YJ. Glutamate in peripheral organs: biology and pharmacology. Eur J Pharmacol. 2016;784:42–48. doi: 10.1016/j.ejphar.2016.05.009. 27164423

[44] Chen Z, He X, Yao MW, et al. [Research advances on the cholinergic inflammatory reflex and inflammation resolution]. Zhonghua Shao Shang Za Zhi. 2021;37(9):885–889. doi: 10.3760/cma.j.cn501120-20200609-00299. 34645156 PMC11917218

[45] Sanders VM. The role of adrenoceptor-mediated signals in the modulation of lymphocyte function. Adv Neuroimmunol. 1995;5(3):283–298. doi: 10.1016/0960-5428(95)00019-x. 8748072

[46] Sun Z, Hou D, Liu S, et al. Norepinephrine inhibits the cytotoxicity of NK92‑MI cells via the β2‑adrenoceptor/cAMP/PKA/p‑CREB signaling pathway. Mol Med Rep. 2018;17(6):8530–8535. doi: 10.3892/mmr.2018.8872. 29658580

[47] Araujo LP, Maricato JT, Guereschi MG, et al. The sympathetic nervous system mitigates CNS autoimmunity via β2-adrenergic receptor signaling in immune cells. Cell Rep. 2019;28(12):3120–3130.e5. doi: 10.1016/j.celrep.2019.08.042. 31533035

[48] Li D, Wu J, Xiong X. The role of the acetylcholine system in common respiratory diseases and COVID-19. Molecules. 2023;28(3):1139. doi: 10.3390/molecules28031139. 36770805 PMC9920988

[49] Huang Y, Zhao C, Su X. Neuroimmune regulation of lung infection and inflammation. QJM. 2019;112(7):483–487. doi: 10.1093/qjmed/hcy154. 30016504

[50] Galle-Treger L, Suzuki Y, Patel N, et al. Nicotinic acetylcholine receptor agonist attenuates ILC2-dependent airway hyperreactivity. Nat Commun. 2016;7(1):13202. doi: 10.1038/ncomms13202. 27752043 PMC5071851

[51] Branchfield K, Nantie L, Verheyden JM, et al. Pulmonary neuroendocrine cells function as airway sensors to control lung immune response. Science (1979). 2016;351(6274):707–710. doi: 10.1126/science.aad7969. PMC4860346 26743624

[52] Barrios J, Patel KR, Aven L, et al. Early life allergen-induced mucus overproduction requires augmented neural stimulation of pulmonary neuroendocrine cell secretion. FASEB J. 2017;31(9):4117–4128. doi: 10.1096/fj.201700115R. 28566470 PMC5572694

[53] Nagashima H, Mahlakõiv T, Shih HY, et al. Neuropeptide CGRP limits group 2 innate lymphoid cell responses and constrains type 2 inflammation. Immunity. 2019;51(4):682–695.e6. doi: 10.1016/j.immuni.2019.06.009. 31353223 PMC6801073

[54] Pinho-Ribeiro FA, Verri WA, Jr, Chiu IM. Nociceptor sensory neuron-immune interactions in pain and inflammation. Trends Immunol. 2017;38(1):5–19. doi: 10.1016/j.it.2016.10.001. 27793571 PMC5205568

[55] Schaible HG. Nociceptive neurons detect cytokines in arthritis. Arthritis Res Ther. 2014;16(5):470. doi: 10.1186/s13075-014-0470-8. 25606597 PMC4289196

[56] Donnelly CR, Chen O, Ji RR. How do sensory neurons sense danger signals? Trends Neurosci. 2020;43(10):822–838. doi: 10.1016/j.tins.2020.07.008. 32839001 PMC7530006

[57] Bhandage AK, Barragan A. GABAergic signaling by cells of the immune system: more the rule than the exception. Cell Mol Life Sci. 2021;78(15):5667–5679. doi: 10.1007/s00018-021-03881-z. 34152447 PMC8316187

[58] Nie Z, Maung JN, Jacoby DB, et al. Lung eosinophils increase vagus nerve-mediated airway reflex bronchoconstriction in mice. Am J Physiol Lung Cell Mol Physiol. 2020;318(2):L242–l251. doi: 10.1152/ajplung.00040.2019. 31746625 PMC7052679

[59] Drake MG, Scott GD, Blum ED, et al. Eosinophils increase airway sensory nerve density in mice and in human asthma. Sci Transl Med. 2018;10(457):eaar8477. doi: 10.1126/scitranslmed.aar8477. 30185653 PMC6592848

[60] Nie Z, Nelson CS, Jacoby DB, et al. Expression and regulation of intercellular adhesion molecule-1 on airway parasympathetic nerves. J Allergy Clin Immunol. 2007;119(6):1415–1422. doi: 10.1016/j.jaci.2007.03.005. 17418379

[61] Toyoshima S, Okayama Y. Neuro-allergology: mast cell-nerve cross-talk. Allergol Int. 2022;71(3):288–293. doi: 10.1016/j.alit.2022.04.002. 35688775

[62] Atta AA, Ibrahim WW, Mohamed AF, et al. Microglia polarization in nociplastic pain: mechanisms and perspectives. Inflammopharmacology. 2023;31(3):1053–1067. doi: 10.1007/s10787-023-01216-x. 37069462 PMC10229465

[63] Charlton T, Prowse N, McFee A, et al. Brain-derived neurotrophic factor (BDNF) has direct anti-inflammatory effects on microglia. Front Cell Neurosci. 2023;17:1188672. doi: 10.3389/fncel.2023.1188672. 37404293 PMC10315457

[64] Gao YJ, Ji RR. Chemokines, neuronal-glial interactions, and central processing of neuropathic pain. Pharmacol Ther. 2010;126(1):56–68. doi: 10.1016/j.pharmthera.2010.01.002. 20117131 PMC2839017

[65] Odoardi F, Sie C, Streyl K, et al. T cells become licensed in the lung to enter the central nervous system. Nature. 2012;488(7413):675–679. doi: 10.1038/nature11337. 22914092

[66] Song Q, E S, Zhang Z, et al. Neuroplasticity in the transition from acute to chronic pain. Neurotherapeutics. 2024;21(6):e00464. doi: 10.1016/j.neurot.2024.e00464. 39438166 PMC11585895

[67] Yirmiya R, Goshen I. Immune modulation of learning, memory, neural plasticity and neurogenesis. Brain Behav Immun. 2011;25(2):181–213. doi: 10.1016/j.bbi.2010.10.015. 20970492

[68] Gonzalez-Rey E, Ganea D, Delgado M. Neuropeptides: keeping the balance between pathogen immunity and immune tolerance. Curr Opin Pharmacol. 2010;10(4):473–481. doi: 10.1016/j.coph.2010.03.003. 20399708 PMC3417345

[69] Nussbaum JC, Van Dyken SJ, von Moltke J, et al. Type 2 innate lymphoid cells control eosinophil homeostasis. Nature. 2013;502(7470):245–248. doi: 10.1038/nature12526. 24037376 PMC3795960

[70] Talbot S, Abdulnour RE, Burkett PR, et al. Silencing nociceptor neurons reduces allergic airway inflammation. Neuron. 2015;87(2):341–354. doi: 10.1016/j.neuron.2015.06.007. 26119026 PMC4506220

[71] Wallrapp A, Riesenfeld SJ, Burkett PR, et al. The neuropeptide NMU amplifies ILC2-driven allergic lung inflammation. Nature. 2017;549(7672):351–356. doi: 10.1038/nature24029. 28902842 PMC5746044

[72] Wiersinga WJ, Rhodes A, Cheng AC, et al. Pathophysiology, transmission, diagnosis, and treatment of coronavirus disease 2019 (COVID-19): a review. JAMA. 2020;324(8):782–793. doi: 10.1001/jama.2020.12839. 32648899

[73] Tong SY, Davis JS, Eichenberger E, et al. Staphylococcus aureus infections: epidemiology, pathophysiology, clinical manifestations, and management. Clin Microbiol Rev. 2015;28(3):603–661. doi: 10.1128/cmr.00134-14. 26016486 PMC4451395

[74] Liu T, Yang L, Han X, et al. Local sympathetic innervations modulate the lung innate immune responses. Sci Adv. 2020;6(20):eaay1497. doi: 10.1126/sciadv.aay1497. 32426489 PMC7220323

[75] Baral P, Umans BD, Li L, et al. Nociceptor sensory neurons suppress neutrophil and γδ T cell responses in bacterial lung infections and lethal pneumonia. Nat Med. 2018;24(4):417–426. doi: 10.1038/nm.4501. 29505031 PMC6263165

[76] Zhu F, Yu D, Qin X, et al. The neuropeptide CGRP enters the macrophage cytosol to suppress the NLRP3 inflammasome during pulmonary infection. Cell Mol Immunol. 2023;20(3):264–276. doi: 10.1038/s41423-022-00968-w. 36600053 PMC9970963

[77] Kwon MJ, Shin HY, Cui Y, et al. CCL2 mediates neuron-macrophage interactions to drive proregenerative macrophage activation following preconditioning injury. J Neurosci. 2015;35(48):15934–15947. doi: 10.1523/jneurosci.1924-15.2015. 26631474 PMC6605453

[78] Guan Z, Kuhn JA, Wang X, et al. Injured sensory neuron-derived CSF1 induces microglial proliferation and DAP12-dependent pain. Nat Neurosci. 2016;19(1):94–101. doi: 10.1038/nn.4189. 26642091 PMC4703328

[79] Cavanaugh DJ, Chesler AT, Bráz JM, et al. Restriction of transient receptor potential vanilloid-1 to the peptidergic subset of primary afferent neurons follows its developmental downregulation in nonpeptidergic neurons. J Neurosci. 2011;31(28):10119–10127. doi: 10.1523/jneurosci.1299-11.2011. 21752988 PMC3147010

[80] Shields SD, Ahn HS, Yang Y, et al. Nav1.8 expression is not restricted to nociceptors in mouse peripheral nervous system. Pain. 2012;153(10):2017–2030. doi: 10.1016/j.pain.2012.04.022. 22703890

[81] Abrahamsen B, Zhao J, Asante CO, et al. The cell and molecular basis of mechanical, cold, and inflammatory pain. Science (1979). 2008;321(5889):702–705. doi: 10.1126/science.1156916. 18669863

[82] Asthma group of Chinese Throacic Society. [Guidelines for bronchial asthma prevent and management(2020 edition) Asthma group of Chinese Throacic Society]. Zhonghua Jie He He Hu Xi Za Zhi. 2020;43(12):1023–1048. doi: 10.3760/cma.j.cn112147-20200618-00721. 33333637

[83] Papi A, Brightling C, Pedersen SE, et al. Asthma. Lancet. 2018;391(10122):783–800. doi: 10.1016/s0140-6736(17)33311-1. 29273246

[84] Lambrecht BN, Hammad H. The immunology of asthma. Nat Immunol. 2015;16(1):45–56. doi: 10.1038/ni.3049. 25521684

[85] Foster PS, Maltby S, Rosenberg HF, et al. Modeling T(H) 2 responses and airway inflammation to understand fundamental mechanisms regulating the pathogenesis of asthma. Immunol Rev. 2017;278(1):20–40. doi: 10.1111/imr.12549. 28658543 PMC5492958

[86] Jean EE, Good O, Rico JMI, et al. Neuroimmune regulatory networks of the airway mucosa in allergic inflammatory disease. J Leukoc Biol. 2022;111(1):209–221. doi: 10.1002/jlb.3ru0121-023r. 33857344 PMC8674821

[87] Sui P, Wiesner DL, Xu J, et al. Pulmonary neuroendocrine cells amplify allergic asthma responses. Science (1979). 2018;360(6393):eaan8546. doi: 10.1126/science.aan8546. PMC6387886 29599193

[88] Kabata H, Moro K, Koyasu S. The group 2 innate lymphoid cell (ILC2) regulatory network and its underlying mechanisms. Immunol Rev. 2018;286(1):37–52. doi: 10.1111/imr.12706. 30294963

[89] Ednacot EMQ, Nabhani A, Dinh DM, et al. Pharmacological potential of cyclic nucleotide signaling in immunity. Pharmacol Ther. 2024;258:108653. doi: 10.1016/j.pharmthera.2024.108653. 38679204

[90] Halim TY, Steer CA, Mathä L, et al. Group 2 innate lymphoid cells are critical for the initiation of adaptive T helper 2 cell-mediated allergic lung inflammation. Immunity. 2014;40(3):425–435. doi: 10.1016/j.immuni.2014.01.011. 24613091 PMC4210641

[91] Ju X, Nagashima A, Dvorkin-Gheva A, et al. Neuromedin-U mediates rapid activation of airway group 2 innate lymphoid cells in mild asthma. Am J Respir Crit Care Med. 2024;210(6):755–765. doi: 10.1164/rccm.202311-2164OC. 38598774

[92] Dragunas G, Woest ME, Nijboer S, et al. Cholinergic neuroplasticity in asthma driven by TrkB signaling. FASEB J. 2020;34(6):7703–7717. doi: 10.1096/fj.202000170R. 32277855 PMC7302963

[93] Kistemaker LEM, Prakash YS. Airway innervation and plasticity in asthma. Physiology (Bethesda). 2019;34(4):283–298. doi: 10.1152/physiol.00050.2018. 31165683 PMC6863372

[94] Cao Y, Li Y, Wang X, et al. Dopamine inhibits group 2 innate lymphoid cell-driven allergic lung inflammation by dampening mitochondrial activity. Immunity. 2023;56(2):320–335.e9. doi: 10.1016/j.immuni.2022.12.017. 36693372

[95] Wang W, Garcia C, Shao F, et al. Lung dopaminergic nerves facilitate the establishment of T(H)2 resident memory cells in early life. J Allergy Clin Immunol. 2023;152(2):386–399. doi: 10.1016/j.jaci.2023.02.011. 36841266 PMC10440294

[96] Ning K, Jiang L, Hu T, et al. ATP-sensitive potassium channels mediate the cardioprotective effect of panax notoginseng saponins against myocardial ischaemia-reperfusion injury and inflammatory reaction. Biomed Res Int. 2020;2020(1):3039184. doi: 10.1155/2020/3039184. 33134375 PMC7593753

[97] Thompson RJ, Sayers I, Kuokkanen K, et al. Purinergic receptors in the airways: potential therapeutic targets for asthma? Front Allergy. 2021;2:677677. doi: 10.3389/falgy.2021.677677. 35386996 PMC8974712

[98] Ferrari D, Vuerich M, Casciano F, et al. Eosinophils and purinergic signaling in health and disease. Front Immunol. 2020;11:1339. doi: 10.3389/fimmu.2020.01339. 32733449 PMC7360723

[99] Gao ZG, Jacobson KA. Purinergic signaling in mast cell degranulation and asthma. Front Pharmacol. 2017;8:947. doi: 10.3389/fphar.2017.00947. 29311944 PMC5744008

[100] Silva-Vilches C, Ring S, Mahnke K. ATP, and its metabolite adenosine as regulators of dendritic cell activity. Front Immunol. 2018;9:2581. doi: 10.3389/fimmu.2018.02581. 30473700 PMC6237882

[101] Li R, Shang Y, Hu X, et al. ATP/P2X7r axis mediates the pathological process of allergic asthma by inducing M2 polarization of alveolar macrophages. Exp Cell Res. 2020;386(1):111708. doi: 10.1016/j.yexcr.2019.111708. 31682811

[102] Kim K, Kim HJ, Binas B, et al. Inflammatory mediators ATP and S100A12 activate the NLRP3 inflammasome to induce MUC5AC production in airway epithelial cells. Biochem Biophys Res Commun. 2018;503(2):657–664. doi: 10.1016/j.bbrc.2018.06.057. 29906464

[103] Vicente CT, Revez JA, Ferreira MAR. Lessons from ten years of genome-wide association studies of asthma. Clin Transl Immunology. 2017;6(12):e165. doi: 10.1038/cti.2017.54. 29333270 PMC5750453

[104] Pompe E, Strand M, van Rikxoort EM, et al. Five-year progression of emphysema and air trapping at CT in smokers with and those without chronic obstructive pulmonary disease: results from the COPDGene study. Radiology. 2020;295(1):218–226. doi: 10.1148/radiol.2020191429. 32013794 PMC7104704

[105] Spiesshoefer J, Regmi B, Ottaviani MM, et al. Sympathetic and vagal nerve activity in COPD: pathophysiology, presumed determinants and underappreciated therapeutic potential. Front Physiol. 2022;13:919422. doi: 10.3389/fphys.2022.919422. 35845993 PMC9281604

[106] Chhabra SK, Gupta M, Ramaswamy S, et al. Cardiac sympathetic dominance and systemic inflammation in COPD. COPD. 2015;12(5):552–559. doi: 10.3109/15412555.2014.974743. 25495489

[107] Vanzella LM, Bernardo AFB, Carvalho TD, et al. Complexity of autonomic nervous system function in individuals with COPD. J Bras Pneumol. 2018;44(1):24–30. doi: 10.1590/s1806-37562017000000086. 29538539 PMC6104537

[108] Su X, Matthay MA, Malik AB. Requisite role of the cholinergic alpha7 nicotinic acetylcholine receptor pathway in suppressing Gram-negative sepsis-induced acute lung inflammatory injury. J Immunol. 2010;184(1):401–410. doi: 10.4049/jimmunol.0901808. 19949071 PMC2877486

[109] Gross NJ, Skorodin MS. Role of the parasympathetic system in airway obstruction due to emphysema. N Engl J Med. 1984;311(7):421–425. doi: 10.1056/nejm198408163110701. 6749189

[110] Calzetta L, Ritondo BL, Zappa MC, et al. The impact of long-acting muscarinic antagonists on mucus hypersecretion and cough in chronic obstructive pulmonary disease: a systematic review. Eur Respir Rev. 2022;31(164):210196. doi: 10.1183/16000617.0196-2021. 35508331 PMC9488979

[111] Noguchi M, Furukawa KT, Morimoto M. Pulmonary neuroendocrine cells: physiology, tissue homeostasis and disease. Dis Model Mech. 2020;13(12):dmm046920. doi: 10.1242/dmm.046920. 33355253 PMC7774893

[112] Barnes PJ, Burney PG, Silverman EK, et al. Chronic obstructive pulmonary disease. Nat Rev Dis Primers. 2015;1(1):15076. doi: 10.1038/nrdp.2015.76. 27189863

[113] Kuo CS, Krasnow MA. Formation of a neurosensory organ by epithelial cell slithering. Cell. 2015;163(2):394–405. doi: 10.1016/j.cell.2015.09.021. 26435104 PMC4597318

[114] Gu X, Karp PH, Brody SL, et al. Chemosensory functions for pulmonary neuroendocrine cells. Am J Respir Cell Mol Biol. 2014;50(3):637–646. doi: 10.1165/rcmb.2013-0199OC. 24134460 PMC4068934

[115] Aguayo SM, King TE Jr, Waldron JA Jr, et al. Increased pulmonary neuroendocrine cells with bombesin-like immunoreactivity in adult patients with eosinophilic granuloma. J Clin Invest. 1990;86(3):838–844. doi: 10.1172/jci114782. 2394833 PMC296800

[116] Aguayo SM, Miller YE, Waldron JA, Jr., et al. Brief report: idiopathic diffuse hyperplasia of pulmonary neuroendocrine cells and airways disease. N Engl J Med. 1992;327(18):1285–1288. doi: 10.1056/nejm199210293271806. 1406819

[117] Stabile A, Pistilli A, Crispoltoni L, et al. A role for NGF and its receptors TrKA and p75NTR in the progression of COPD. Biol Chem. 2016;397(2):157–163. doi: 10.1515/hsz-2015-0208. 26408608

[118] Li L, Kong L, Fang X, et al. SH2-B beta expression in alveolar macrophages in BAL fluid of asthmatic guinea pigs and its role in NGF-TrkA-mediated asthma. Respirology. 2009;14(1):60–68. doi: 10.1111/j.1440-1843.2008.01417.x. 19144050

[119] Burian B, Angela S, Nadler B, et al. Inhaled vasoactive intestinal peptide (VIP) improves the 6-minute walk test and quality of life in patients with COPD: the VIP/COPD-trial. Chest. 2006;130(4):121S. doi: 10.1378/chest.130.4_MeetingAbstracts.121S-c.

[120] Paulis L, Rajkovicova R, Simko F. New developments in the pharmacological treatment of hypertension: dead-end or a glimmer at the horizon? Curr Hypertens Rep. 2015;17(6):557. doi: 10.1007/s11906-015-0557-x. 25893478 PMC4412646

[121] Cai J, Chen Q, Mehrabi Nasab E, et al. Immunomodulatory effect of N-acetyl-seryl-aspartyl-proline and vasoactive intestinal peptide on chronic obstructive pulmonary disease pathophysiology. Fundam Clin Pharmacol. 2022;36(6):1005–1010. doi: 10.1111/fcp.12811. 35763864

[122] Selvarajah B, Platé M, Chambers RC. Pulmonary fibrosis: emerging diagnostic and therapeutic strategies. Mol Aspects Med. 2023;94:101227. doi: 10.1016/j.mam.2023.101227. 38000335

[123] Wang Y, Wang X, Du C, et al. Glycolysis and beyond in glucose metabolism: exploring pulmonary fibrosis at the metabolic crossroads. Front Endocrinol (Lausanne). 2024;15:1379521. doi: 10.3389/fendo.2024.1379521. 38854692 PMC11157045

[124] Sgalla G, Iovene B, Calvello M, et al. Idiopathic pulmonary fibrosis: pathogenesis and management. Respir Res. 2018;19(1):32. doi: 10.1186/s12931-018-0730-2. 29471816 PMC5824456

[125] Hinz B, Lagares D. Evasion of apoptosis by myofibroblasts: a hallmark of fibrotic diseases. Nat Rev Rheumatol. 2020;16(1):11–31. doi: 10.1038/s41584-019-0324-5. 31792399 PMC7913072

[126] Liu SS, Liu C, Lv XX, et al. The chemokine CCL1 triggers an AMFR-SPRY1 pathway that promotes differentiation of lung fibroblasts into myofibroblasts and drives pulmonary fibrosis. Immunity. 2021;54(9):2042–2056.e8. doi: 10.1016/j.immuni.2021.06.008. 34407391

[127] Zhang K, Yao E, Wang SA, et al. A functional circuit formed by the autonomic nerves and myofibroblasts controls mammalian alveolar formation for gas exchange. Dev Cell. 2022;57(13):1566–1581.e7. doi: 10.1016/j.devcel.2022.05.021. 35714603 PMC9308505

[128] Li D, Guabiraba R, Besnard AG, et al. IL-33 promotes ST2-dependent lung fibrosis by the induction of alternatively activated macrophages and innate lymphoid cells in mice. J Allergy Clin Immunol. 2014;134(6):1422–1432.e11. doi: 10.1016/j.jaci.2014.05.011. 24985397 PMC4258609

[129] Otaki N, Motomura Y, Terooatea T, et al. Activation of ILC2s through constitutive IFNγ signaling reduction leads to spontaneous pulmonary fibrosis. Nat Commun. 2023;14(1):8120. doi: 10.1038/s41467-023-43336-6. 38097562 PMC10721793

[130] Moriyama S, Brestoff JR, Flamar AL, et al. β(2)-adrenergic receptor-mediated negative regulation of group 2 innate lymphoid cell responses. Science (1979). 2018;359(6379):1056–1061. doi: 10.1126/science.aan4829. 29496881

[131] Cardoso V, Chesné J, Ribeiro H, et al. Neuronal regulation of type 2 innate lymphoid cells via neuromedin U. Nature. 2017;549(7671):277–281. doi: 10.1038/nature23469. 28869974 PMC5714273

[132] Klose CSN, Mahlakõiv T, Moeller JB, et al. The neuropeptide neuromedin U stimulates innate lymphoid cells and type 2 inflammation. Nature. 2017;549(7671):282–286. doi: 10.1038/nature23676. 28869965 PMC6066372

[133] Wu X, Zhou X, Hu Y, et al. Neutralization of nerve growth factor (NGF) inhibits the Th2 response and protects against the respiratory syncytial virus (RSV) infection. Immunol Res. 2017;65(3):721–728. doi: 10.1007/s12026-017-8909-z. 28258348

[134] Liu P, Li S, Tang L. Nerve growth factor: A potential therapeutic target for lung diseases. Int J Mol Sci. 2021;22(17):9112. doi: 10.3390/ijms22179112. 34502019 PMC8430922

[135] Szema AM, Forsyth E, Ying B, et al. NFATc3 and VIP in idiopathic pulmonary fibrosis and chronic obstructive pulmonary disease. PLoS One. 2017;12(1):e0170606. doi: 10.1371/journal.pone.0170606. 28125639 PMC5270325

[136] Duan JX, Guan XX, Yang HH, et al. Vasoactive intestinal peptide attenuates bleomycin-induced murine pulmonary fibrosis by inhibiting epithelial-mesenchymal transition: restoring autophagy in alveolar epithelial cells. Int Immunopharmacol. 2021;101(Pt B):108211. doi: 10.1016/j.intimp.2021.108211. 34634687

[137] Zhang YF, Zhang J, Sun CC, et al. Vasoactive intestinal peptide inhibits the activation of murine fibroblasts and expression of interleukin 17 receptor C. Cell Biol Int. 2019;43(7):770–780. doi: 10.1002/cbin.11151. 31026365

[138] Itano J, Taniguchi A, Senoo S, et al. Neuropeptide Y antagonizes development of pulmonary fibrosis through IL-1β inhibition. Am J Respir Cell Mol Biol. 2022;67(6):654–665. doi: 10.1165/rcmb.2021-0542OC. 36122332

[139] Xu J, Xu F. Role of neurogenic substance P in overexpression of alveolar macrophages’ neurokinin 1 receptor in mice exposed to cigarette smoke. Exp Lung Res. 2010;36(4):243–254. doi: 10.3109/01902140903398275. 20426532

[140] Xu J, Xu F, Lin Y. Cigarette smoke synergizes lipopolysaccharide-induced interleukin-1β and tumor necrosis factor-α secretion from macrophages via substance P-mediated nuclear factor-κB activation. Am J Respir Cell Mol Biol. 2011;44(3):302–308. doi: 10.1165/rcmb.2009-0288OC. 20160043 PMC3095931

[141] Mo C, Li H, Yan M, et al. Dopaminylation of endothelial TPI1 suppresses ferroptotic angiocrine signals to promote lung regeneration over fibrosis. Cell Metab. 2024;36(8):1839–1857.e12. doi: 10.1016/j.cmet.2024.07.008. 39111287

[142] Gao R, Peng X, Perry C, et al. Macrophage-derived netrin-1 drives adrenergic nerve-associated lung fibrosis. J Clin Invest. 2021;131(1):e136542. doi: 10.1172/jci136542. PMC7773383 33393489

[143] Barnaby D, Ferrick K, Kaplan DT, et al. Heart rate variability in emergency department patients with sepsis. Acad Emerg Med. 2002;9(7):661–670. doi: 10.1111/j.1553-2712.2002.tb02143.x. 12093705

[144] Huston JM, Tracey KJ. The pulse of inflammation: heart rate variability, the cholinergic anti-inflammatory pathway, and implications for therapy. J Intern Med. 2011;269(1):45–53. doi: 10.1111/j.1365-2796.2010.02321.x. 21158977 PMC4527046

[145] Sung H, Ferlay J, Siegel RL, et al. Global cancer statistics 2020: GLOBOCAN estimates of incidence and mortality worldwide for 36 cancers in 185 countries. CA Cancer J Clin. 2021;71(3):209–249. doi: 10.3322/caac.21660. 33538338

[146] Gadepalli VS, Deb SP, Deb S, et al. Lung cancer stem cells, p53 mutations, and MDM2. Subcell Biochem. 2014;85:359–370. doi: 10.1007/978-94-017-9211-0_19. 25201204

[147] Ganti AK, Klein AB, Cotarla I, et al. Update of incidence, prevalence, survival, and initial treatment in patients with non-small cell lung cancer in the US. JAMA Oncol. 2021;7(12):1824–1832. doi: 10.1001/jamaoncol.2021.4932. 34673888 PMC8532041

[148] Jiang SH, Hu LP, Wang X, et al. Neurotransmitters: emerging targets in cancer. Oncogene. 2020;39(3):503–515. doi: 10.1038/s41388-019-1006-0. 31527667

[149] Bujak JK, Kosmala D, Szopa IM, et al. Inflammation, cancer and immunity-implication of TRPV1 channel. Front Oncol. 2019;9:1087. doi: 10.3389/fonc.2019.01087. 31681615 PMC6805766

[150] White JP, Urban L, Nagy I. TRPV1 function in health and disease. Curr Pharm Biotechnol. 2011;12(1):130–144. doi: 10.2174/138920111793937844. 20932253

[151] Zahalka AH, Frenette PS. Nerves in cancer. Nat Rev Cancer. 2020;20(3):143–157. doi: 10.1038/s41568-019-0237-2. 31974491 PMC7709871

[152] Duitama M, Moreno Y, Santander SP, et al. TRP channels as molecular targets to relieve cancer pain. Biomolecules. 2021;12(1):1. doi: 10.3390/biom12010001. 35053150 PMC8774023

[153] Entschladen F, Drell T, Lang K, et al. Tumour-cell migration, invasion, and metastasis: navigation by neurotransmitters. Lancet Oncol. 2004;5(4):254–258. doi: 10.1016/s1470-2045(04)01431-7. 15050959

[154] Wang Y, Zhang Y, Ouyang J, et al. TRPV1 inhibition suppresses non-small cell lung cancer progression by inhibiting tumour growth and enhancing the immune response. Cell Oncol (Dordr). 2024;47(3):779–791. doi: 10.1007/s13402-023-00894-7. 37902941 PMC12973945

[155] Zhang B, Vogelzang A, Miyajima M, et al. B cell-derived GABA elicits IL-10(+) macrophages to limit anti-tumour immunity. Nature. 2021;599(7885):471–476. doi: 10.1038/s41586-021-04082-1. 34732892 PMC8599023

[156] Furrukh M. Tobacco smoking and lung cancer: perception-changing facts. Sultan Qaboos Univ Med J. 2013;13(3):345–358. doi: 10.12816/0003255. 23984018 PMC3749017

[157] Zoli M, Pucci S, Vilella A, et al. Neuronal and extraneuronal nicotinic acetylcholine receptors. Curr Neuropharmacol. 2018;16(4):338–349. doi: 10.2174/1570159x15666170912110450. 28901280 PMC6018187

[158] Song P, Sekhon HS, Lu A, et al. M3 muscarinic receptor antagonists inhibit small cell lung carcinoma growth and mitogen-activated protein kinase phosphorylation induced by acetylcholine secretion. Cancer Res. 2007;67(8):3936–3944. doi: 10.1158/0008-5472.Can-06-2484. 17440109

[159] Lin G, Sun L, Wang R, et al. Overexpression of muscarinic receptor 3 promotes metastasis and predicts poor prognosis in non-small-cell lung cancer. J Thorac Oncol. 2014;9(2):170–178. doi: 10.1097/jto.0000000000000066. 24419413 PMC4132044

[160] Xu R, Shang C, Zhao J, et al. Activation of M3 muscarinic receptor by acetylcholine promotes non-small cell lung cancer cell proliferation and invasion via EGFR/PI3K/AKT pathway. Tumour Biol. 2015;36(6):4091–4100. doi: 10.1007/s13277-014-2911-z. 25964092

[161] Zhao Q, Yue J, Zhang C, et al. Inactivation of M2 AChR/NF-κB signaling axis reverses epithelial-mesenchymal transition (EMT) and suppresses migration and invasion in non-small cell lung cancer (NSCLC). Oncotarget. 2015;6(30):29335–29346. doi: 10.18632/oncotarget.5004. 26336823 PMC4745730

[162] Hirsch FR, Scagliotti GV, Mulshine JL, et al. Lung cancer: current therapies and new targeted treatments. Lancet. 2017;389(10066):299–311. doi: 10.1016/s0140-6736(16)30958-8. 27574741

[163] Nie M, Chen N, Pang H, et al. Targeting acetylcholine signaling modulates persistent drug tolerance in EGFR-mutant lung cancer and impedes tumor relapse. J Clin Invest. 2022;132(20):e160152. doi: 10.1172/jci160152. PMC9566900 36048538

[164] Cox MA, Duncan GS, Lin GHY, et al. Choline acetyltransferase-expressing T cells are required to control chronic viral infection. Science (1979). 2019;363(6427):639–644. doi: 10.1126/science.aau9072. PMC7181845 30733420

[165] Mashimo M, Moriwaki Y, Misawa H, et al. Regulation of immune functions by non-neuronal acetylcholine (ACh) via muscarinic and nicotinic ACh receptors. Int J Mol Sci. 2021;22(13):6818. doi: 10.3390/ijms22136818. 34202925 PMC8268711

[166] Khanmammadova N, Islam S, Sharma P, et al. Neuro-immune interactions and immuno-oncology. Trends Cancer. 2023;9(8):636–649. doi: 10.1016/j.trecan.2023.05.002. 37258398 PMC10524972

[167] Casibang M, Purdom S, Jakowlew S, et al. Prostaglandin E2 and vasoactive intestinal peptide increase vascular endothelial cell growth factor mRNAs in lung cancer cells. Lung Cancer. 2001;31(2-3):203–212. doi: 10.1016/s0169-5002(00)00168-9. 11165399

[168] Moody TW, Walters J, Casibang M, et al. VPAC1 receptors and lung cancer. Ann N Y Acad Sci. 2000;921(1):26–32. doi: 10.1111/j.1749-6632.2000.tb06947.x. 11193832 PMC8820160

[169] Cha JE, Bae WY, Choi JS, et al. Angiogenic activities are increased via upregulation of HIF-1α expression in gefitinib-resistant non-small cell lung carcinoma cells. Oncol Lett. 2021;22(3):671. doi: 10.3892/ol.2021.12932. 34345296 PMC8323004

[170] D’Amico AG, Maugeri G, Rasà DM, et al. Modulatory role of PACAP and VIP on HIFs expression in lung adenocarcinoma. Peptides. 2021;146:170672. doi: 10.1016/j.peptides.2021.170672. 34627957

[171] Wehkamp U, Stern S, Krüger S, et al. Co-expression of NGF and PD-L1 on tumor-associated immune cells in the microenvironment of Merkel cell carcinoma. J Cancer Res Clin Oncol. 2018;144(7):1301–1308. doi: 10.1007/s00432-018-2657-x. 29744662 PMC11813430

[172] Griffin N, Faulkner S, Jobling P, et al. Targeting neurotrophin signaling in cancer: the renaissance. Pharmacol Res. 2018;135:12–17. doi: 10.1016/j.phrs.2018.07.019. 30031169

[173] Wijaya LK, Stumbles PA, Drummond PD. Tumor necrosis factor α induces α(1B)-adrenergic receptor expression in keratinocytes. Cytokine. 2020;125:154851. doi: 10.1016/j.cyto.2019.154851. 31520851

[174] Gao F, Griffin N, Faulkner S, et al. The neurotrophic tyrosine kinase receptor TrkA and its ligand NGF are increased in squamous cell carcinomas of the lung. Sci Rep. 2018;8(1):8135. doi: 10.1038/s41598-018-26408-2. 29802376 PMC5970205

[175] Yang C, Sun Y, Ouyang X, et al. Pain may promote tumor progression via substance P-dependent modulation of toll-like receptor-4. Pain Med. 2020;21(12):3443–3450. doi: 10.1093/pm/pnaa265. 32914185

[176] Ahmed A, Wang JH, Redmond HP. Silencing of TLR4 increases tumor progression and lung metastasis in a murine model of breast cancer. Ann Surg Oncol. 2013;20 Suppl 3(S3):S389–S396. doi: 10.1245/s10434-012-2595-9. 22890596

[177] Beyer K, Partecke LI, Roetz F, et al. LPS promotes resistance to TRAIL-induced apoptosis in pancreatic cancer. Infect Agent Cancer. 2017;12(1):30. doi: 10.1186/s13027-017-0139-4. 28572836 PMC5450120

[178] Luo XZ, He QZ, Wang K. Expression of Toll-like receptor 4 in ovarian serous adenocarcinoma and correlation with clinical stage and pathological grade. Int J Clin Exp Med. 2015;8(8):14323–14327. doi: 10.1136/jclinpath-2015-203160. 26550417 PMC4613102

[179] Kazarian M, Laird-Offringa IA. Small-cell lung cancer-associated autoantibodies: potential applications to cancer diagnosis, early detection, and therapy. Mol Cancer. 2011;10(1):33. doi: 10.1186/1476-4598-10-33. 21450098 PMC3080347

[180] Honnorat J, Cartalat-Carel S, Ricard D, et al. Onco-neural antibodies and tumour type determine survival and neurological symptoms in paraneoplastic neurological syndromes with Hu or CV2/CRMP5 antibodies. J Neurol Neurosurg Psychiatry. 2009;80(4):412–416. doi: 10.1136/jnnp.2007.138016. 18931014 PMC2664637

[181] Gandhi L, Johnson BE. Paraneoplastic syndromes associated with small cell lung cancer. J Natl Compr Canc Netw. 2006;4(6):631–638. doi: 10.6004/jnccn.2006.0052. 16813730

[182] Mohamed MZ, Abed El Baky MF, Ali ME, et al. Aprepitant exerts anti-fibrotic effect via inhibition of TGF-β/Smad3 pathway in bleomycin-induced pulmonary fibrosis in rats. Environ Toxicol Pharmacol. 2022;95:103940. doi: 10.1016/j.etap.2022.103940. 35931359

[183] Kuronuma K, Otsuka M, Wakabayashi M, et al. Role of transient receptor potential vanilloid 4 in therapeutic antifibrotic effects of pirfenidone. Am J Physiol Lung Cell Mol Physiol. 2022;323(2):L193–L205. doi: 10.1152/ajplung.00565.2020. 35787697

[184] Nourinezhad J, Tabrizinejad MN, Janeczek M. Detailed gross anatomy and topography of the sympathetic cardiac nerves and related ganglia in Syrian hamsters (Mesocricetus auratus). Ann Anat. 2022;239:151842. doi: 10.1016/j.aanat.2021.151842. 34673201

[185] Ma W, Dumont Y, Vercauteren F, et al. Lipopolysaccharide induces calcitonin gene-related peptide in the RAW264.7 macrophage cell line. Immunology. 2010;130(3):399–409. doi: 10.1111/j.1365-2567.2009.03239.x. 20141542 PMC2913219

[186] Jia Y, Lee LY. Role of TRPV receptors in respiratory diseases. Biochim Biophys Acta. 2007;1772(8):915–927. doi: 10.1016/j.bbadis.2007.01.013. 17346945

[187] McLeod RL, Correll CC, Jia Y, et al. TRPV1 antagonists as potential antitussive agents. Lung. 2008;186 Suppl 1(S1):S59–S65. doi: 10.1007/s00408-007-9032-z. 17926096

[188] Gavva NR, Treanor JJ, Garami A, et al. Pharmacological blockade of the vanilloid receptor TRPV1 elicits marked hyperthermia in humans. Pain. 2008;136(1-2):202–210. doi: 10.1016/j.pain.2008.01.024. 18337008

[189] Ferkol T, Schraufnagel D. The global burden of respiratory disease. Ann Am Thorac Soc. 2014;11(3):404–406. doi: 10.1513/AnnalsATS.201311-405PS. 24673696

[190] Global burden of chronic respiratory diseases and risk factors, 1990-2019: an update from the Global Burden of Disease Study 2019. EClinicalMedicine. 2023;59:101936. doi: 10.1016/j.eclinm.2023.101936. 37229504 PMC7614570

[191] Hvizdos KM, Goa KL. Tiotropium bromide. Drugs. 2002;62(8):1195–1203; discussion 1204-5. doi: 10.2165/00003495-200262080-00008. 12010082

[192] Lebold KM, Drake MG, Hales-Beck LB, et al. IL-5 exposure in utero increases lung nerve density and airway reactivity in adult offspring. Am J Respir Cell Mol Biol. 2020;62(4):493–502. doi: 10.1165/rcmb.2019-0214OC. 31821769 PMC7110978

[193] Ortega HG, Liu MC, Pavord ID, et al. Mepolizumab treatment in patients with severe eosinophilic asthma. N Engl J Med. 2014;371(13):1198–1207. doi: 10.1056/NEJMoa1403290. 25199059

[194] Kum E, Patel M, Diab N, et al. Efficacy and tolerability of gefapixant for treatment of refractory or unexplained chronic cough: A systematic review and dose-response meta-analysis. JAMA. 2023;330(14):1359–1369. doi: 10.1001/jama.2023.18035. 37694849 PMC10495930

[195] Matera MG, Rogliani P, Page CP, et al. The discovery and development of gefapixant as a novel antitussive therapy. Expert Opin Drug Discov. 2024;19(10):1159–1172. doi: 10.1080/17460441.2024.2391902. 39138872

[196] Li W, Zhu H, Zou X, et al. A brain-to-lung signal from GABAergic neurons to ADRB2(+) interstitial macrophages promotes pulmonary inflammatory responses. Immunity. 2025;58(8):2069–2085.e9. doi: 10.1016/j.immuni.2025.05.005. 40466637

[197] Kong J, Wen S, Cao W, et al. Lung organoids, useful tools for investigating epithelial repair after lung injury. Stem Cell Res Ther. 2021;12(1):95. doi: 10.1186/s13287-021-02172-5. 33516265 PMC7846910

[198] Saorin G, Caligiuri I, Rizzolio F. Microfluidic organoids-on-a-chip: the future of human models. Semin Cell Dev Biol. 2023;144:41–54. doi: 10.1016/j.semcdb.2022.10.001. 36241560

[199] Lees RM, Pichler B, Packer AM. Contribution of optical resolution to the spatial precision of two-photon optogenetic photostimulation in vivo. Neurophotonics. 2024;11(1):015006. doi: 10.1117/1.NPh.11.1.015006. 38322022 PMC10846536

[200] Chen H, Deng C, Gao J, et al. Integrative spatial analysis reveals tumor heterogeneity and immune colony niche related to clinical outcomes in small cell lung cancer. Cancer Cell. 2025;43(3):519–536.e5. 15. doi: 10.1016/j.ccell.2025.01.012. 39983726

[201] Shi F, Ergashev A, Pan Z, et al. Macrophage-mimicking nanotherapy for attenuation of acute pancreatitis. Mater Today Bio. 2025;30:101406. doi: 10.1016/j.mtbio.2024.101406. PMC11733200 39816666

[202] Hosang L, Canals RC, van der Flier FJ, et al. The lung microbiome regulates brain autoimmunity. Nature. 2022;603(7899):138–144. doi: 10.1038/s41586-022-04427-4. 35197636

[203] Tao B, Li X, Hao M, et al. Organoid-guided precision medicine: from bench to bedside. MedComm (2020). 2025;6(5):e70195. doi: 10.1002/mco2.70195. 40321594 PMC12046123

[204] Saperstein S, Chen L, Oakes D, et al. IL-1beta augments TNF-alpha-mediated inflammatory responses from lung epithelial cells. J Interferon Cytokine Res. 2009;29(5):273–284. doi: 10.1089/jir.2008.0076. 19231998 PMC2718541

